# Tissue-preferential recruitment of electron transfer chains for cytochrome P450-catalyzed phenolic biosynthesis

**DOI:** 10.1126/sciadv.ade4389

**Published:** 2023-01-11

**Authors:** Xianhai Zhao, Yunjun Zhao, Mingyue Gou, Chang-Jun Liu

**Affiliations:** Biology Department, Brookhaven National Laboratory, Upton, NY 11973, USA.

## Abstract

Cytochrome P450 system consists of P450 monooxygenase and redox pattern(s). While the importance of monooxygenases in plant metabolism is well documented, the metabolic roles of the related redox components have been largely overlooked. Here, we show that distinct electron transfer chains are recruited in phenylpropanoid-monolignol P450 systems to support the synthesis and distribution of different classes of phenolics in different plant tissues. While *Arabidopsis* cinnamate 4-hydroxylase adopts conventional NADPH-cytochrome P450 oxidoreductase (CPR) electron transfer chain for its *para*-hydroxylation reaction, ferulate 5-hydroxylase uses both NADPH-CPR-cytochrome *b_5_* (CB5) and NADH–cytochrome *b_5_* reductase–CB5 chains to support benzene ring 5-hydroxylation, in which the former route is primarily recruited in the stem for syringyl lignin synthesis, while the latter dominates in the syntheses of 5-hydroxylated phenolics in seeds and seed coat suberin. Our study unveils an additional layer of complexity and versatility of P450 system that the plants evolved for diversifying phenolic repertoires.

## Teaser

Phenylpropanoid P450 system employs distinct electron transfer chains to synthesize distinct phenolics in different plant tissues.

## INTRODUCTION

As sessile organism, land plants evolve robust and versatile metabolic capacity to produce a variety of specialized metabolites to cope with the harsh terrestrial ecosystem. One of the remarkable adaptation events is the expansion of cytochrome P450 superfamily enzymes, which leads to the generation of amazing chemical diversity (*1*, *2*). P450s are a ubiquitous superfamily of heme-thiolate enzymes that catalyze mixed-function monooxygenase reactions. They are crucial to the biosynthesis and metabolism of fatty acids, phytosterols, plant growth regulators, and a variety of plant specialized metabolites, including phenylpropanoids, terpenoids, glucosinolates, indolalkaloid phytoalexins, etc. (*1*, *2*). Eukaryotic microsomal P450 system contains two essential components, the cytochrome P450 monooxygenase and the related redox partner(s). In each catalytic cycle, two electrons as reducing power, donated from redox partner(s) to the P450 prosthetic center, are required for inserting a molecule of activated oxygen into a hydrophobic substrate (*3*). The endoplasmic reticula (ER) membrane of eukaryotic cells contains two electron transfer systems: the reduced form of nicotinamide adenine dinucleotide (NADH)–dependent system consisting of NADH–cytochrome *b_5_* reductase (CBR) and cytochrome *b_5_* (CB5) protein; the reduced form of nicotinamide adenine dinucleotide phosphate (NADPH)–dependent system containing NADPH–cytochrome P450 reductase (CPR). The former transfers reducing equivalents from the pyridine nucleotide cofactor NADH first to CB5 intermediate and then to the terminal acceptors including P450s, while the latter directly transfers electrons from the cofactor NADPH to P450 enzymes (fig. S1). In microsomal P450 system, CPR has been regarded as the typical electron donor. It interacts with cognate P450 enzymes via electron static and transfers both electrons directly from NADPH to the P450 catalytic center (*4*). Evidence from mammalian P450 systems also reveal that NADPH-dependent CPR can reduce CB5 (*5*, *6*). In such case, CB5 hypothetically delivers the second electron from CPR to the P450 ferrous-O_2_ complex. A small set of P450-catalyzed reactions in xenobiotics and drug metabolism are affected with CB5 proteins, in which CB5 shows either stimulation or inhibition effects (*6*, *7*). A couple of studies have also suggested that CB5 proteins are involved in plant specialized metabolisms (*8*, *9*). Nevertheless, it remains unclear how this electron shuttle protein participates in the electron transfer system(s) to support P450-catalyzed reactions.

Differing from yeast or mammalian endomembrane system, where only one copy of *CPR*, *CBR*, or *CB5* gene exists, the higher plant genomes in general contain multiple gene copies encoding the redox components. For instance, *Arabidopsis thaliana* has two true genes, termed as *ARABIDOPSIS THALIANA P450-REDUCTASE 1 and 2* (*ATR1* and *ATR2*), encoding two ER-resident CPRs (*10*). Both the recombinant ATRs are fully functional in transferring reducing equivalents from NADPH to plant P450s, and their biochemical functions and enzymatic properties are indistinguishable in heterologous experimental system (*11*, *12*). However, two *ATR* genes display distinct expression patterns in *Arabidopsis*, with *ATR2* more in lignifying tissues and being induced with lignin biosynthesis and by wounding and light treatment, while *ATR1* more constitutively expressed in all the examined tissues (*10*). Disruption of *ATR2* resulted in ~6% total lignin reduction and the notable composition shift from syringyl (S)–lignin subunits to hydroxyphenyl (H)–lignin subunits (*10*). Analogous to *CPRs*, two annotated *CBRs* exist in *A. thaliana* but only the encoded CBR1 flavoprotein is the ER resident and involved in the microsomal electron transfer system (*13*). Depletion of *CBR1* decreases 18:3 unsaturated fatty acid in seeds and affects pollen function, fertilization, and seed maturation (*13*), indicating the involvement of this redox protein in fatty acid desaturation. The encoded second *Arabidopsis* CBR, CBR2, is localized to the inner mitochondrial membrane, and no evidence shows that it is part of the ER electron transport system (*14*). The ER-resident CBR1 transfers electrons from reductants to its flavin adenine dinucleotide prosthetic group and subsequently to the heme of CB5. *Arabidopsis* CBR1 displays strict specificity to NADH for reducing CB5, it has no detectable catalytic activity when NADPH is used as reductant. Similarly, *Arabidopsis* CPR stringently uses NADPH to reduce CB5 proteins (*11*, *15*). Sharply contrast to the single copy of *CB5* gene in mammals where it generates two CB5 isoforms via alternative splicing, with one located to the ER membrane and the other in cytoplasm (*6*, *16*), the higher plants including *Arabidopsis* evolve multiple copies of *CB5s* (*15*, *17*–*20*). Five CB5 members and one CB5-like protein are encoded by *A. thaliana* genome, termed as AtCB5A to AtCB5E and CB5LP (*20*). Among them, AtCB5B, AtCB5C, AtCB5D, and AtCB5E share higher sequence similarity at their amino acid levels and localize to the ER membrane (*8*, *18*, *20*), implicating their involvement in the microsomal electron transfer systems. Similar to that in mammals, the ER-resident CB5 proteins conventionally are implicated in the lipid desaturation and long-chain fatty acid elongation (*19*, *21*). Nevertheless, our recent study reveals that one of the *Arabidopsis* CB5 isoforms, AtCB5D, is an indispensable electron donor functionally associated with monolignol biosynthetic P450 enzyme ferulate 5-hydroxylase (F5H) to support S-lignin biosynthesis (*8*). Disruption of *AtCB5D* resulted in severe reduction of S-lignin content in mature stem and the 5-hydroxylated phenolic esters in *Arabidopsis* leaves (*8*). However, it is not clear in which electron transfer system(s) that AtCB5D is involved for phenolic biosynthesis and what metabolic roles the electron transfer systems might play.

Starting with phenylalanine, phenylpropanoid metabolism generates C6-C3 core structure that elaborates a vast variety of aromatic compounds, including the methanolic soluble flavonoids/anthocyanins, stilbenes, coumarins, hydroxycinnamate esters, and lignans and the intractable polymers suberin, proanthocyanidins, and lignin (*22*). These simple and polymeric phenolics function as photoprotectant, phytoalexin, antifeedant, or physical barrier, playing vital roles in plant growth, development, and environmental interaction. Diverged from general phenylpropanoid pathway, monolignol branch directs the majority of phenylpropanoid flux to the production of three hydroxycinnamyl alcohols (monolignols), i.e., *p*-coumaryl alcohol, coniferyl alcohol, and sinapyl alcohol, which are further polymerized through radical coupling in the cell walls of vascular cells, forming heteropolymer lignin (fig. S2). As a structural component, lignin provides compressive strength and hydrophobic environment to conducting tissues to facilitate water transport and acts as a mechanical barrier preventing phytopathogen infection (*23*). The structural subunits of lignin derived from three monolignols are commonly referred to as hydroxyphenyl (H), guaiacyl (G), and syringyl (S) lignin, respectively. While H- and G-lignin are fundamental to all the tracheophytes, S-lignin is specific to certain lineages, e.g., flowering plants and the lycophyte *Selaginella* (*24*).

Concomitant with monolignol biosynthesis, the biosynthetic intermediates such as *p*-coumaric, ferulic, and sinapic acids can conjugate with long-chain fatty acids to form lipophilic polyester suberin in particular tissues, such as seed coat and root endodermis, which controls water, solute, and gas movement (fig. S2) (*25*). In addition, these phenolics can also be transformed to a variety of vacuolar stored phenolic esters, e.g., the antifeedant sinapoylcholine (sinapine) dominant in seeds and the ultraviolet (UV)–protectant sinapoyl malate primarily accumulated in leaf epidermis of *Brassicaceae* species (fig. S2) (*26*, *27*).

In phenylpropanoid-monolignol biosynthetic pathway, three hydroxylation steps occur on the benzene ring of phenolics. These are the 4-, 3′-, and 5-hydroxylations catalyzed by cinnamic acid 4-hydroxylase (C4H; CYP73A5), *p*-coumaroyl ester 3′-hydroxylase 1 (C3′H1; CYP98A3), and F5H1 (CYP84A1), respectively, in *Arabidopsi*s (fig. S2) (*28*–*30*). C4H catalyzes 4-hydroxylation of the first aromatic compound cinnamic acid in general phenylpropanoid pathway, producing central intermediate for the entire class of phenolics. C3′H1 as a key monolignol branch-point enzyme hydroxylates *p*-coumaroyl ester derivatives at the phenyl ring 3′-position, leading to the formation of G- and S-lignin monomers. F5H1 hydroxylates coniferyl alcohol/aldehyde at its benzene ring 5-position, specific for S monolignol formation in angiosperms. F5H1 activity also determines the synthesis of 5-hydroxylated derivatives sinapoyl esters found in the *Brassicaceae* family (fig. S2). C4H and C3′H1 are believed to deviate from their ancient primary metabolic cousins, for example, sterol 14-demethylase, thus representing the early evolution events in the invention of phenolic synthesis (*31*); whereas the emergence of F5H1 in angiosperms is regarded as a recent evolutionary event along with the invention of S-lignin branch in the flowering plants (*32*).

The specific roles of monolignol P450s in the synthesis of lignin and the related phenolics are well determined. Down-regulation or elimination of those P450s alters lignin content and composition and/or retards plant growth and development (*28*, *30*, *33*). These metabolic and physiological impacts of plant P450 systems are commonly attributed to the terminal P450 enzymes, the redox partners/electron transfer components associated with the system are conventionally regarded as auxiliary elements, and their metabolic roles have been largely ignored. In the present study, with yeast whole-cell biocatalytic assays, the in vitro microsomal activity determination, and the in planta genetic exploration, we discover that different electron transfer components and their constituted electron transport chains are differentially involved in the biosynthesis of distinct classes of phenolics. Specifically, monolignol biosynthetic C4H and F5H1 couple with distinct electron transfer systems to support their catalysis; furthermore, the synthesis and distribution of different classes of 5-hydroxylated phenolic derivatives, catalyzed by the same F5H1 enzyme, use different electron transfer chains in *Arabidopsis* stem, leaf, seed, and seed coat tissues. Therefore, electron donor systems, in addition to P450 enzymes themselves, constitute an additional layer of regulatory mechanism, contributing to the metabolic diversity of phenylpropanoids.

## RESULTS

### CB5 proteins exhibit functional specificity for S-lignin biosynthesis

Previously, we found that the *Arabidopsi*s CB5 family member, AtCB5D, acts as an obligate electron carrier supporting AtF5H1-catalyzed benzene ring 5-hydroxlation for S-lignin synthesis (*8*). *Arabidopsis* genome encodes five canonical CB5 proteins including the ER-resident AtCB5B, AtCB5C, AtCB5D, and AtCB5E (*20*). To assess whether those canonical CB5s are involved in phenylpropanoid-lignin biosynthesis, the homozygous transfer DNA (T-DNA) insertion mutants *cb5a-1* (SALKseq_054962), *cb5b-1* (SALK_100161), *cb5c-1* (SALK_027748), *cb5d-1* (SALK_045010), and *cb5e-1* (SALK_151509) were obtained (fig. S3A). Reverse transcription-quantitative polymerase chain reaction (RT-qPCR) validation indicated that *cb5a-1*, *cb5c-1*, and *cb5d-1* represent the null knockout mutants, where their gene expression levels were undetectable or negligible, whereas *cb5b-1* with a T-DNA insertion at 3′ untranslated region and *cb5e-1* with a T-DNA insertion in 5′ untranslated region of the genes remained a portion of gene expression, representing the knockdown mutants (fig. S3B). Subsequently, the higher order mutants were generated through genetic cross of the obtained single mutant lines. Because *AtCB5B* and *AtCB5C* are located on the chromosome 2 and *AtCB5D* and *AtCB5E* on chromosome 5, homozygous double mutants *cb5bc* and *cb5de* were first generated, denoted as *bc* and *de*, respectively. Because the T-DNA insertion line *cb5c-1* was reported to generate a mutant with an insertion within the coding sequence of the *AtCB5C* gene that results in a recombinant protein with 39 new C-terminal residues, which likely causes mislocalization of the protein, thus might not reflecting the true biological function of AtCB5C (*34*), CRISPR-Cas9 strategy was therefore applied to knock out *AtCB5C* gene in the *cb5b-1* background to create the double mutant *cb5b-1 cb5c-cr1* (*bc-cr1*) (fig. S3C). A homozygous quadruple mutant *bcde* was then obtained by genetic cross of *bc-cr1* with *de* double mutant.

**Fig. 1.**
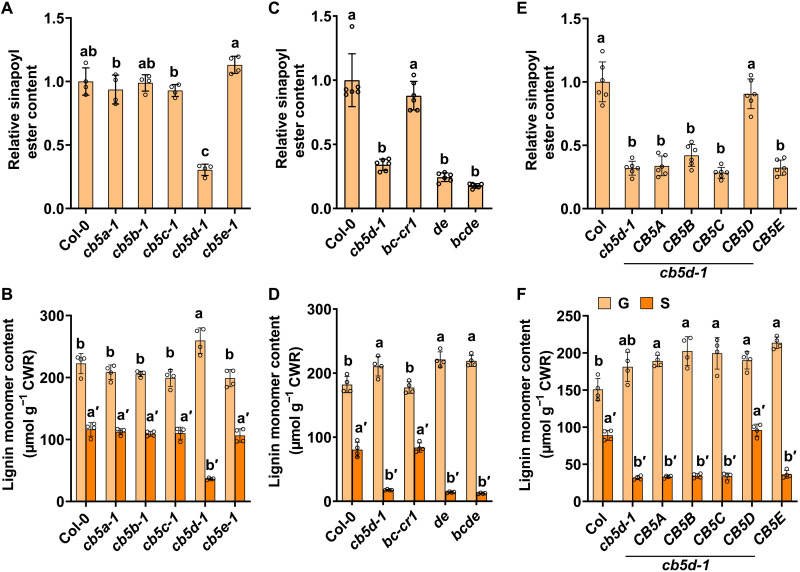
Accumulation of sinapoyl esters and lignin in *cb5* mutants and complementation plants. (**A** and **C**) Relative content of sinapoyl esters in the 4-week-old rosette leaves of *cb5* single (A), double, and quadruple (C) mutants. The level of the Col-0 WT was set as 1. (**B** and **D**) Lignin monomer contents released by thioacidolysis in the 11-week-old stems of *cb5* single (B), double, and quadruple (D) mutants. Two rosette leaves from different plants were mixed representing one biology replicate for sinapoyl ester analysis. Six stems were pooled representing one biology replicate for lignin thioacidolysis analysis. (**E** and **F**) Complementation of *cb5d-1* with five *Arabidopsis CB5* genes. The 4-week-old leaves and 11-week-old stems of the primary transgenic plants were analyzed for sinapoyl esters (E) and lignin monomers (F), respectively. Two rosette leaves from different plants were mixed representing one biology replicate for sinapoyl ester analysis. Two to four stems were pooled representing one biology replicate for lignin thioacidolysis analysis. Data are presented as means ± SD of four (A, B, D, and F) or six (C and E) biological replicates. Small letters on the bars indicate significant differences (*P* < 0.05), as determined by one-way analysis of variance (ANOVA) test. CWR, cell wall residues.

The soluble phenolic esters and lignin content and composition were analyzed with the 4-week-old rosette leaves and the 11-week-old stems of *cb5* mutants, respectively. Consistent with the previous study (*8*), disruption of *AtCB5D* alone resulted in ~70% reduction of the accumulation level of leaf sinapoyl esters and 69% decrease of stem S-lignin subunits, while G-lignin subunits were conversely slightly increased, compared with the wild-type (WT) controls (Fig. 1, A and B). However, disruption of other *CB5* genes did not result in obvious impairment in the accumulation of either leaf sinapoyl esters or stem lignin composition (Fig. 1, A and B). The same did the double mutant *bc-cr1* (Fig. 1, C and D), while the double mutant *de* essentially resembled the *cb5d-1* (Fig. 1, C and D). Even in the quadruple mutant *bcde*, the levels of leaf sinapoyl esters and stem S-lignin monomer showed only slight but insignificant decrease compared to those in the *cb5d-1* single and *de* double mutants (Fig. 1, C and D). These data indicate that AtCB5D plays a dominant role in supporting leaf phenolics and stem lignin biosynthesis, and the CB5 members exhibit little functional redundancy.

To further confirm the contributions of CB5 members in leaf sinapoyl esters and stem S-lignin biosynthesis, *AtCB5A*, *AtCB5B*, *AtCB5C*, *AtCB5D*, and *AtCB5E* genes, driven by *AtC4H* promoter, were respectively introduced into *cb5d-1* mutant. Except *AtCB5D* itself, none of the other *CB5* transgenes rescued the defects of *cb5d-1* in synthesis of leaf sinapoyl esters and stem S-lignin (Fig. 1, E and F). Together, these data suggest that CB5 proteins have specialized functions in planta, where AtCB5D is evolved for primarily supporting F5H-catalyzed reaction for the synthesis of 5-hydroxylated derivatives sinapoyl esters and S-lignin monomers.

Analogous to *cb5d-1*, the double and quadruple mutants *de* and *bcde* showed a higher level of G-lignin monomers compared to the WT (Fig. 1D) and a similar level of total lignin content as well as a normal growth as the WT (fig. S4). These data suggest that all the ER-resident CB5 proteins have no direct functional association with C4H- and/or C3′H-catalyzed reactions that lead to the biosynthesis of G-lignin.

### Both CBR and ATR reduce CB5D

**Fig. 2.**
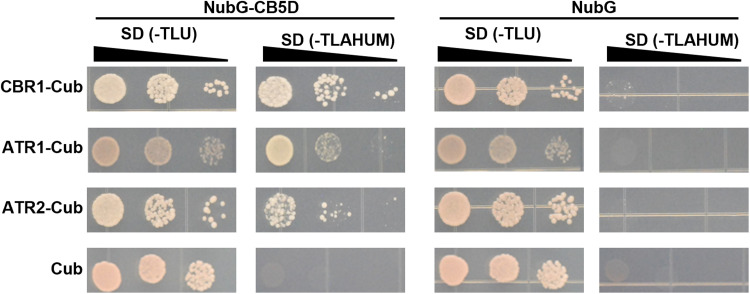
Physical interaction between CB5D and CBR1, ATR1, or ATR2 in yeast. CBR1, ATR1, and ATR2 were used as baits. CB5D was used as prey. After a series of dilution (10^−1^ to 10^−3^), yeast diploid cells harboring both bait and prey were plated on the growth [SD/-Trp (T), -Leu (L), and -Ura (U)] and selective [SD/-Trp, -Leu, -Ade (A), -His (H), -Ura, -Met and (M)] media, as indicated. Yeast cells harboring the empty vector Cub and/or NubG were used as the controls. Images were captured after incubation for 3 days at 28°C. Experiments were repeated twice, and the similar results were obtained.

**Fig. 3.**
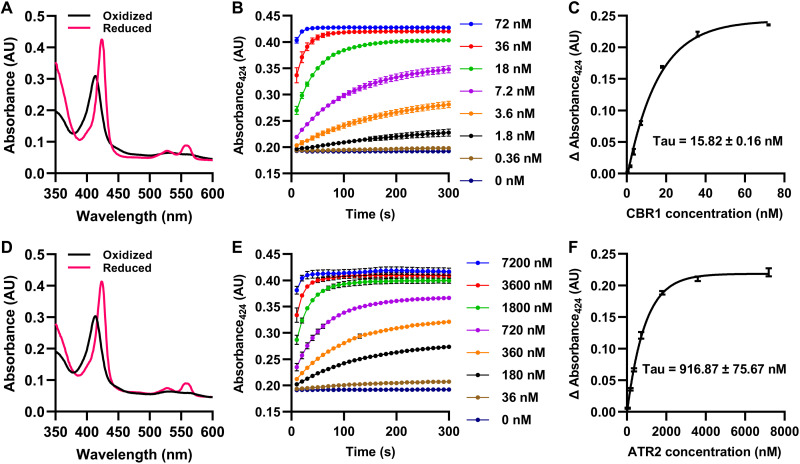
The reduction of CB5D by CBR1 and ATR2. The recombinant CB5D (7 μM) was reduced by different concentrations of CBR1 (**A** to **C**) or ATR2 (**D** to **F**) at room temperature. The reduction reaction was initiated by addition of either 100 μM NADH (A to C) or 100 μM NADPH (D to F) as an electron donor. (A and D) Absolute absorption spectra of the oxidized (blank) and the completely reduced (red) CB5D proteins before and after treatment with 72 nM CBR1 (A) or 7200 nM ATR2 (D). (B and E) Changes of absorbance at 424 nm of CB5D over the indicated period of reaction with different concentrations of CBR1 (B) or ATR2 (E). The absorption was recorded for 30 cycles with a 10-s interval. (C and F) Monophasic kinetics of CB5D reduction by CBR1 (C) or ATR2 (F) at the point of 60-s reaction at the function of different concentrations of the reductase. Data are presented as means ± SD of three independent experiments. Tau is the concentration constant. AU, absorbance units.

CB5 protein is known to be reduced by CBR and CPR (*6*). Testing the potential physical interactions between AtCB5D and *Arabidopsis* redox proteins CBR1, ATR1, and ATR2 using the split ubiquitin membrane yeast two-hybrid system, we found that yeast cells harboring AtCB5D and each of the tested reductases grew well on the selective media, indicative of their physical interactions (Fig. 2). These data suggest that AtCB5D might serve as the electron acceptor substrate for both CBR and CPR. We then reconstituted NADH-CBR and NADPH-ATR electron transfer systems using the recombinant CBR1 and ATR2 enzymes with removal of their N-terminal transmembrane domains (fig. S5A). The truncated recombinant AtCB5D with removal of its C-terminal transmembrane domain (fig. S5) was used as the electron acceptor to spectroscopically monitor its reduction rates, i.e., the electron transfer rate from NADH or NADPH to CB5 protein. Cytochrome C (Cyt C) was included in the experiments as an authentic control set, because it is an artificial substrate for CPR and, in some cases, CBR also reduces it directly (*35*). Both the recombinant CBR1 and ATR2 reduced Cyt C effectively, when coupled with NADH or NADPH, respectively (fig. S6), verifying that both recombinant reductases are functional and are enabled to deliver electrons from the reduced pyridine nucleotide cofactors to the acceptor. Moreover, the spectroscopic data showed that ATR2 was more effective than CBR1 to reduce substrate Cyt C, because the required amount of ATR2 to maximally reduce Cyt C was approximately 50 times less than that of CBR1 (fig. S6). Then, the recombinant AtCB5D was examined as the substrate in the same reduction systems. The truncated recombinant AtCB5D spectroscopically displayed an absorption maximum at 413 nm with its oxidized form, and the prominent absorption peaks at 424 and 558 nm with its dithionite reduced form (fig. S5B), indictive of a typical CB5 protein characteristics (*36*). Then, the reduction efficiencies of NADH-CBR1 and NADPH-ATR2 to AtCB5D protein were compared by incubating the same amount of oxidized form of the recombinant AtCB5D with a series of concentrations of the recombinant CBR1 or ATR2 in the presence of 100 μm of NADH or NADPH, respectively (Fig. 3). Similar to Cyt C, AtCB5D was reduced both by NADH-CBR1 and by NADPH-ATR2 at their catalytic concentrations. After 5-min incubation in both reaction systems with the catalytic concentration of CBR1 or ATR2, the oxidized AtCB5D was completely converted into the reduced form, which was indicated with the clear shift of the maximal absorption of AtCB5D from 413 nm of the oxidized form to 424 nm of the reduced protein species (Fig. 3A and D). In sharp contrast to the substrate Cyt C, about 60-fold higher amount of ATR2 than that of CBR1 was required to completely reduce the same amount of oxidized AtCB5D species (Fig. 3, B, C, E, and F). Therefore, CBR1 shows higher catalytic efficiency in reducing AtCB5D than does ATR2. In addition, both *Arabidopsis* CPR isoforms ATR1 and ATR2 exhibited a similar catalytic behavior in reducing AtCB5D (fig. S7). These data suggest that AtCB5D as an electron carrier biochemically more prefers for functional association with CBR over ATR.

### Disruption of *ATR* and *CBR* differentially affects stem S-lignin accumulation

**Fig. 4.**
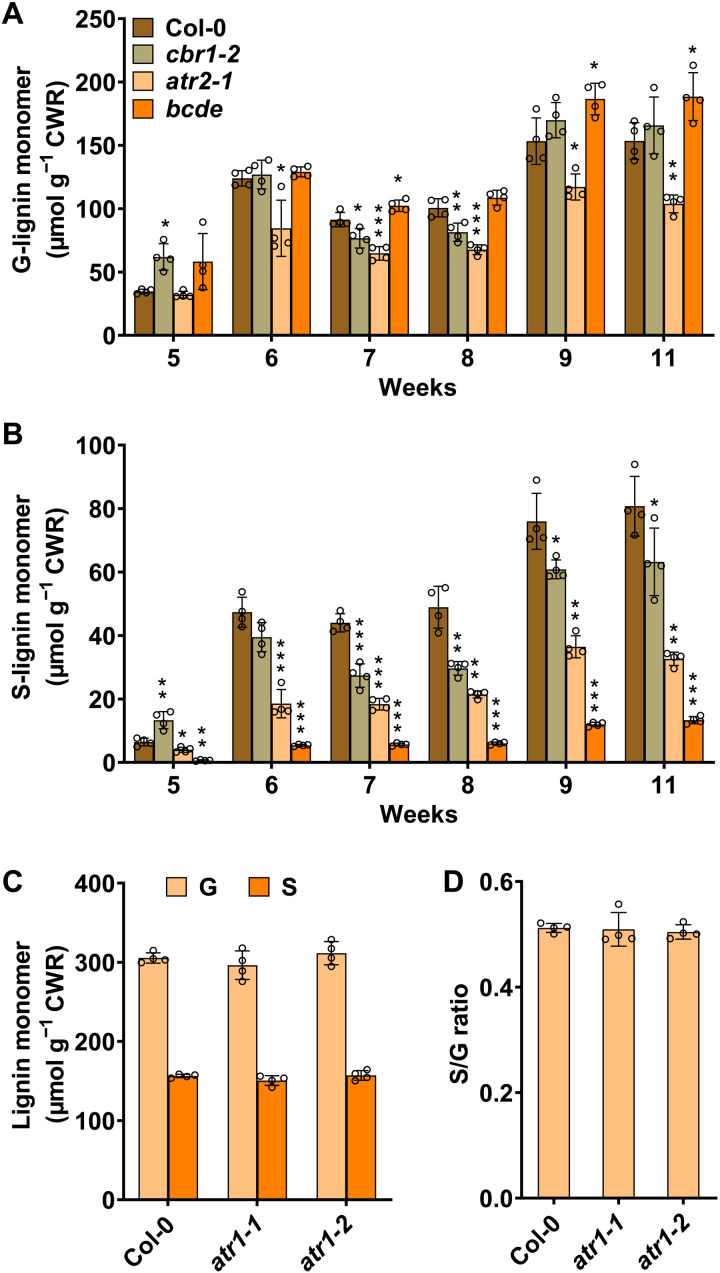
Accumulation of stem lignin monomers during plant development. (**A** and **B**) G- and S-lignin monomer contents, released by thioacidolysis from the stems of indicated developmental stages of the Col-0 WT, *cbr1-2*, *atr2-1*, and *bcde* plants. (**C**) G- and S-lignin monomer contents and (**D**) S/G ratio in the 11-week-old stems of the Col-0, *atr1-1*, and *atr1-2* plants. For all the analyses in (A) to (D), six primary stems were pooled as one biology replicate. Data are presented as means ± SD of four biological replicates. Asterisks indicate significant difference compared to the Col-0 with *P < 0.05, **P < 0.01, and ***P < 0.001 (two-tailed Student’s *t* tests).

Previously, with the fully matured *Arabidopsis* stem, we revealed a negligible effect of CBR1 on stem S-lignin accumulation (*8*). With the observation of NADH-CBR1 effectively reducing AtCB5D in vitro, we revisited *Arabidopsis cbr1-2*, *atr2-1*, and *bcde* mutant lines by examining their stem lignin composition in a course of plant development from 5^th^ to 11^th^ week after they were planted in the soil (Fig. 4). As expected, a constant reduction of G-lignin content was observed in *atr2-1* during the entire course of stem development, compared to the WT control at the same growth stage. In contrast, despite the slight decline in the 7- and 8-week-old stem samples, the G-lignin content in the stem of *cbr1-2* essentially remained unperturbed. In the *CB5* quadruple mutant *bcde*, G-lignin content even displayed a slight increase at the later growth stages (Fig. 4A). Therefore, ATR2 appears to be the only required redox partner functionally associated with C4H and/or C3′H for G-lignin biosynthesis, and CBR1 and CB5s have little or no direct effect on the G-lignin formation.

Examining S-lignin content, we confirmed the marked reduction in both the *atr2-1* and *bcde* lines in all developmental stages, validating the involvement of both ATR2 and CB5 in S-lignin biosynthesis (Fig. 4B). However, only a limited reduction of S-lignin monomer was observed in the *cbr1-2* line after 7 weeks of plant growth, and the level of reduction was much less than that occurred in the *atr2-1* or *bcde* line (Fig. 4B). In the 11-week-old plant stems, *cbr1-2* line showed only ~22% decrease in S-lignin content, while 60 and 83% reductions occurred in the *atr2-1* and *bcde* lines, respectively (Fig. 4B). These data implicate that both NADH-CBR1-CB5 and NADPH-ATR2-CB5 electron pathways participate in the F5H-catalyzed S-lignin synthesis during stem development; nevertheless, the contributions of these two electron delivery pathways are largely different. The CBR1-mediated electron transfer system plays a limited role in the stem S-lignin formation.

Because *Arabidopsis* has two CPR homologous genes, *ATR1* and *ATR2*, and the encoded enzymes have similar catalytic properties (*11*), we also obtained two homozygous T-DNA insertion mutant alleles *atr1-1* (SALK_208483) and *atr1-2* (SALKseq_051672). In both alleles, the *ATR1* gene expression level remains less than 15% (fig. S8). Both G- and S-lignin content and S/G ratio in two *atr1* alleles of the 11-week-old plants, however, did not exhibit obvious alteration, relative to those of the WT (Fig. 4, C and D), suggesting that ATR1 does not participate in or only plays a minor role in stem lignin biosynthesis. Together, these data demonstrate that *Arabidopsis* electron transfer components are differentially involved in lignin biosynthesis. ATR2-CB5 electron transport pathway plays dominant role for stem S-lignin synthesis.

### Accumulation of leaf sinapoyl esters is not perturbed in either *cbr1* or *atr* mutant

*Arabidopsis* leaf epidermis accumulates the 5-methoxylated phenolic esters sinapoyl malate and sinapoyl glucose to protect UV irradiation (*37*). The synthesis of sinapoyl esters necessarily requires AtF5H1 activity and CB5 protein, which were reflected with the nearly complete loss of sinapoyl esters in *fah1* (F5H1 null mutant) (*8*) and up to 80% loss in *bcde* (fig. S9). In contrast, the redox component mutants *atr1-1*, *atr1-2*, *atr2-1*, and *cbr1-2* accumulated almost the same amount of sinapoyl esters in their leaves as that in the WT (fig. S9). These data implicate that the CBR1-, ATR1-, or ATR2-mediated electron transfer systems might redundantly function with AtCB5D and/or F5H1 for the leaf phenolic ester formation.

### Accumulation of seed phenolic esters is compromised primarily in *cbr1*

**Fig. 5.**
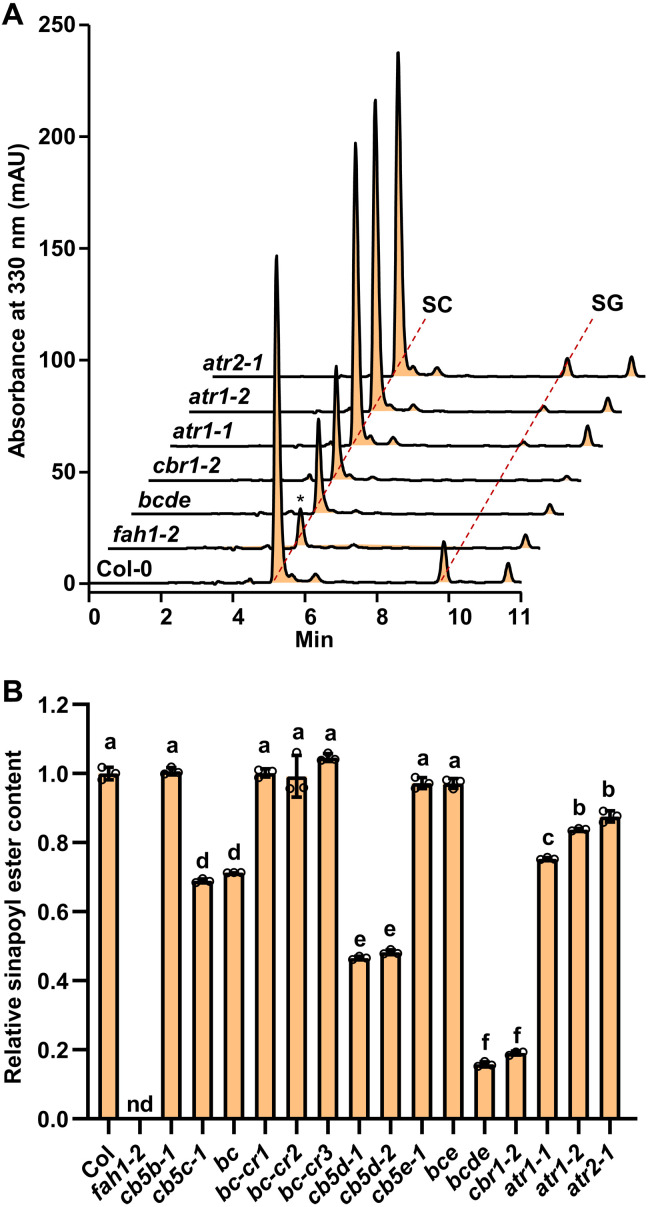
Accumulation of sinapoyl esters in the seeds of the redox component mutants. (**A**) HPLC-UV profiles of methanolic extracts from the mature seeds of Col-0 WT, *fah1-2*, *bcde*, *cbr1-2*, *atr1-1*, *atr1-2*, and *atr2-1* mutants. Seed extracts were prepared with 50% methanol containing 1.5% acetic acid and analyzed by HPLC with UV detection at 330 nm, showing the presence of sinapoylcholine (SC) and sinapoyl glucose (SG). The asterisk-indicated peak at 5 min in *fah1-2* profile represents an undefined background metabolite coresolved with sinapoylcholine, which was confirmed by UHPLC-MS. (**B**) Quantification of seed sinapoyl ester content from the indicated mutant lines. The summed level of both SC and SG of the Col-0 WT was set as 1. Data are presented as means ± SD of three biological replicates. Letters above the bars indicate significant differences (*P* < 0.05), determined by one-way ANOVA test. nd, not detected.

Apart from synthesis of stem lignin and leaf sinapoyl esters, members of *Brassicaceae* family accumulate specific sinapate ester, i.e., sinapoylcholine in seeds, which is considered as a major antinutritive compound in seeds of important crop plants like *Brassica napus* (*26*, *27*). Sinapoylcholine is an alkaloidal amine of the conjugation of sinapic acid with choline. Its synthesis relies on F5H1-catalyzed 5-hdroxylation (*26*). Determining methanolic soluble phenolics extracted from the mature seeds of *Arabidopsis cb5* mutants and *F5H1*-deficient line *fah1-2* with ultra-high performance liquid chromatography–mass spectrometry (UHPLC-MS) (fig. S10), we found that neither sinapoylcholine nor sinapoylglucose was detected in *fah1-2*, and in two *AtCB5D* single mutant alleles, *cb5d-1* and *cb5d-2,* sinapoyl esters were decreased to less than 50% of the WT level (Fig. 5). While down-regulation of *AtCB5B* and *AtCB5E* singly did not impair seed sinapoyl esters accumulation, unexpectedly, the *AtCB5C* T-DNA insertion mutant *cb5c-1* and its genetic cross double mutant with *cb5b*, i.e., *cb5b-1 cb5c-1* (*bc*), showed a reduced level of sinapoyl esters (Fig. 5). However, three *CB5C* knockout lines generated via CRISPR-Cas9 gene editing in *cb5b-1* background, i.e., *bc-cr1*, *bc-cr2*, and *bc-cr3*, where the *CB5C* gene has a 238–base pair (bp) deletion, 1-bp insertion, and 1-bp deletion, respectively (fig. S3C), accumulated the similar level of sinapoyl esters as that of WT (Fig. 5). These data implicate that the impairment effect of *cb5c-1* T-DNA insertion mutation on seed sinapoyl ester accumulation is not from its authentic function, because this T-DNA insertion causes the mutation to produce an abnormal CB5 protein (*34*). Even in the *cb5b-1 cb5c-cr1 cb5e-1* (*bce*) triple mutant, the sinapoyl ester content remained no obvious change relative to that of the WT; until with incorporation of *cb5d-1*, the quadruple mutant *bcde* exhibited a synergistic depletion on seed phenolic accumulation, producing approximately only 16% of the WT level of sinapoyl esters (Fig. 5). These results indicate that similar to the synthesis of stem S-lignin and leaf sinapoyl malate, CB5 and F5H are necessarily required for seed sinapoyl ester formation. Among CB5 proteins, AtCB5D plays a prime role, and the other CB5 proteins have little effect on seed sinapoyl ester synthesis, although some of their transcripts could be detected in the developing seeds (fig. S11).

Determining sinapoyl esters of the *Arabidopsis* mature seeds deficient in *ATR1*, *ATR2*, or *CBR1* genes, respectively, unexpectedly, we found that, in contrast to the observed notable effect of ATR2 on stem S-lignin accumulation, the seed sinapoyl ester content in *atr2-1* showed minor alteration relative to that of the WT; instead, a drastic reduction of sinapoyl ester content occurred in *cbr1-2*, where the total level was reduced more than 81% (Fig. 5). In addition, disruption of *ATR1* caused up to 25% reduction of seed sinapoyl esters, but the effect was much less than that occurred in *cbr1-2* (Fig. 5). These results suggest that both NADH-CBR1-CB5 and NADPH-ATR-CB5 electron transfer chains function with F5H1 for seed sinapoyl ester biosynthesis but quite different from what occurs in the stem S-lignin biosynthesis, the NADH-CBR1-CB5 pathway rather than NADPH-ATR-CB5 chain plays a dominant role in the synthesis of seed phenolics of 5-hydroxylated derivatives.

### Disruption of CBR1 and ATR differentially affects seed coat suberin aromatics

**Fig. 6.**
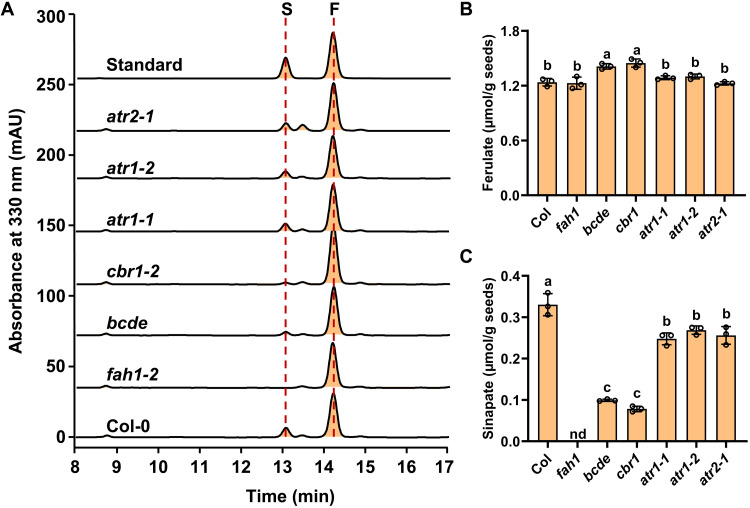
Accumulation of seed coat suberin aromatics in the redox component mutants. (**A**) HPLC-UV profiles of seed coat suberin aromatics from the Col-0 WT, *fah1-2*, *bcde*, *cbr1-2*, *atr11*, *atr1-2*, and *atr2-1* mutants. The extract-free residues of seed coats and an authentic standard composed of ferulic acid and sinapic acid were treated with boron trichloride solution, and the released aromatics were extracted with chloroform. Feruloyl (F) and sinapoyl (S) methylesters were analyzed by HPLC with UV detection at 330 nm. (**B** and **C**) Quantification of the detected suberin ferulate (B) and sinapate (C) in the seed coats of the indicated mutant lines. Data are presented as means ± SD of three biological replicates. Letters above the bars indicate significant differences (*P* < 0.05), determined by one-way ANOVA test.

*Arabidopsis* seed coats are composed of lipophilic polyester suberin, in which the major aromatic components are ferulate and sinapate (*38*). Those ester bond-linked phenolics can be released by transesterification depolymerization of polyesters (*38*, *39*). After removing soluble phenolics, the extractive-free seed coat residues were treated with transesterification reagent boron trichloride/methanol to depolymerize suberin and to release its lipidic monomers and aromatics. The data showed different effects of disruption of electron transfer components on the accumulation of seed coat ferulate and sinapate (Fig. 6). The levels of released ferulate showed no decrease in the seed coats of *cbr1-2*, *bcde*, *atr1-1*, *atr1-2*, and *atr2-1* lines, compared to the WT control. Disruption of *CBR1* and *CB5* resulted in the slight increase of suberin ferulate (Fig. 6, A and B, and fig. S12). In contrast, suberin sinapate content exhibited ~64 and ~ 83% reduction in *bcde* and *cbr1-2* lines, respectively. In addition, ~20% reduction of suberin sinapate occurred in *atr1* and *atr2* alleles (Fig. 6, A and C). These results suggest that similar to the seed-soluble sinapoyl ester synthesis, NADH-CBR1-CB5 electron transfer chain plays primary role in sustaining F5H-catalyzed reaction for seed coat sinapate biosynthesis, but it is not required for the biosynthesis of suberin ferulate. Meanwhile, the redox components ATR1 and ATR2 may redundantly function in seed coat ferulate biosynthesis.

### The genes encoding redox components display differential expression patterns

**Fig. 7.**
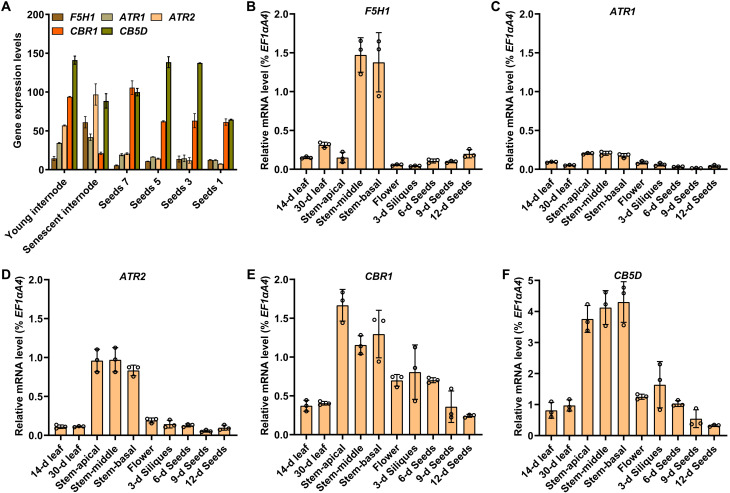
Transcript abundance of *AtF5H1*, *ATR1*, *ATR2*, *CBR1,* and *CB5D* in various *Arabidopsis* tissues. (**A**) In silico gene expression of the color-indicated genes in the stem internodes and seeds. The data were exacted from Klepikova *Arabidopsis* Atlas eFP browser. Young internodes were harvested at the first flower anthesis; senescent internodes were harvested when the first silique turned yellow and was ready to open; the seeds were sampled when the first formed silique was green and 1.5-cm long (*40*). (**B** to **F**) Relative transcript abundance of *AtF5H1* (B), *ATR1* (C), *ATR2* (D), *CBR1* (E), and *CB5D* (F) in the indicated *Arabidopsis* tissues quantified by RT-qPCR. The leaves were from 14-day-old seedlings and 30-day-old plants; the stems and flowers were from 50-day-old plants, the siliques or seeds were collected at the indicated day after anthesis. Tissues from at least five individual plants were pooled as one biological replicate. The expression levels were normalized to that of *EF1*α*A4* gene. Data are presented as means ± SD for three biology replicates.

The differential involvement of electron transfer components ATR1/ATR2, CBR1, and CB5 in phenolics biosynthesis in different tissues promoted us to examine their gene expression patterns. The in silico expression data from Klepikova *Arabidopsis* Atlas Electronic Fluorescent Pictographic (eFP) browser (*40*) showed that At*CB5D* displayed broad and high expression across all the examined developing seeds and the young and matured stem internodes, whereas *ATR2* and *CBR1* exhibited discernible tissue preferential expression patterns, with *CBR1* expressed highly in the developing seeds and young internodes while *ATR2* more preferentially expressed in the senescent stem internodes (Fig. 7A). To further validate the gene expression patterns, we conducted RT-qPCR analysis (Fig. 7, B to F). All the primers used in qPCR were examined for their amplification efficiencies, which showed the values of 94 to 106%, indicative of their optimal amplification (fig. S13). After normalizing with transcripts of housekeeping gene *EF1*α*A4*, the RT-qPCR data confirmed that *AtCB5D*, relative to the other tested redox partner genes, exhibited the most abundant transcription levels across all the examined tissues including leaf, stem, flower, and developing silique. *CBR1* displayed a similar expression pattern as did *AtCB5D*. Both genes showed relatively higher expression in both the stem and developing seed (Fig. 7, E and F), while *ATR2* displayed more preferential expression in the stem but less in the developing seeds, the pattern largely similar to that of *F5H1* (Fig. 7, B and D). *ATR1* exhibited low and constitutive expression across all the examined tissues (Fig. 7C). Both the in silico expression data and the RT-qPCR analysis suggest differential expression patterns of the examined electron transfer components.

### NADPH and NADH show different abundance in *Arabidopsis* tissues

NADH and NADPH are essential reductants involved in many cellular redox processes, including supplying reducing equivalent for CBR- and CPR-mediated electron transfer chains, respectively (*41*). With assumption that the availability of those cofactors might also contribute to the control of synthesis of different phenolics in plant tissues, we measured the in vivo NAD(H) and NADP(H) levels in the developing leaves, stems, and seeds at series of time points of the day via bioluminescent assay. Strong luminescence signals for both NAD(H) and NADP(H) were monitored within the leaf and seed extracts, indicative of their high abundance in the examined tissues; moreover, the overall content of both reductants did not show substantial fluctuation over the examined time periods (fig. S14, A and B). Unexpectedly, the detected overall levels of reductants in the developing stem (per unit of dry weight) were much lower than those determined in the seed and leaf tissues. This is probably due to the lignifying stem tissues contain less number of biochemically active cells per unit of materials. Moreover, he primarily detected reductant in the developing stems was NADP(H); the abundance of NAD(H) was constantly low within the same tissues (fig. S14, C and F). These data indicate that the predominant reducing power in the developing stem is NADPH.

### Reconstructed electron transfer chains differentially support monolignol P450 activities

Knowing the necessity of AtCB5D in supporting of F5H1 but not of C4H (or C3′H) activity in planta and the involvement of different electron transfer components for phenolic synthesis in different tissues, to further validate the functional association of different electron transfer pathways with monolignol biosynthetic P450-catalyzed reactions, we developed a whole-cell biocatalytic system via engineering yeasts with different electron transfer chains. Among three monolignol P450 enzymes, C4H and F5H1 were selected as the representatives. C3′H was not included due to the unavailability of its substrate. We coexpressed *C4H* and *F5H1* with *Arabidopsis* electron transfer components *CB5D*, *CBR1*, or *CPR* (i.e., *ATR1* and *ATR2*) alone or in a combinatory manner, driven by a galactose-inducible promoter, in *Saccharomyces cerevisiae* strain deficient in its endogenous *CPR* (*ncp1*, heterozygous diploids) (*42*), *CB5* (*19*), or *CBR* (*42*) gene. A mutated CB5D, 2muCB5D, with mutations of two histidine residues known to be the key axial ligands for heme binding thus devoid of electron transfer property (*8*), was included as the control. *C4H* and *F5H1* genes, together with the genes encoding a particular electron transfer chain were constructed within the same expression cassette via polycistronic expression strategy, which enables both C4H and F5H1 with their redox partners to express within the same yeast cell and under the exact same experimental conditions thus facilitating a reliable comparison. Before the phenolic substrate feeding, the availability of reductant cofactors NAD(H) and NADP(H) was determined in three yeast experimental strains and confirmed their presence (fig. S14G). After galactose induction for protein expression, yeast cells were fed with *trans*-cinnamic acid, the substrate for C4H, or coniferyl alcohol, the substrate for F5H1. The transformed products were monitored with UHPLC-MS (fig. S15).

**Fig. 8.**
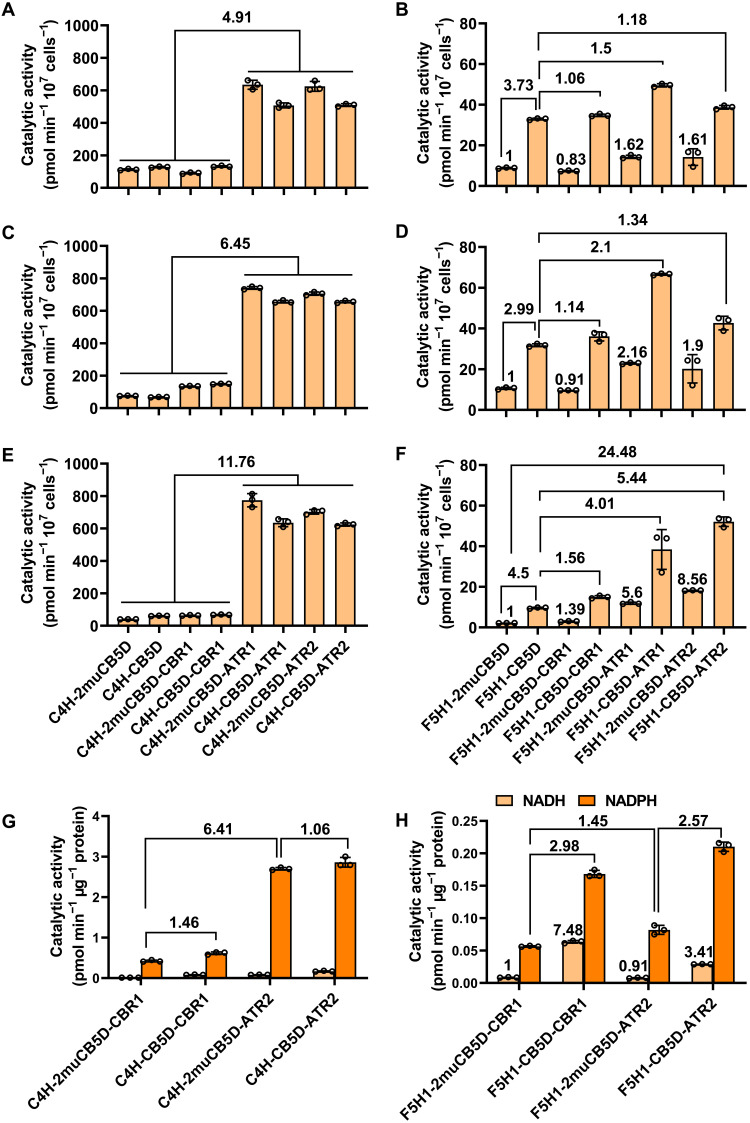
Catalytic activities of C4H and F5H1 in yeast cells harboring the reconstructed electron transfer chains. (**A** to **F**) Whole-cell feeding assays for C4H (A, C, and E) or F5H1 (B, D, and F) activities with the engineered yeast cells deficient in their endogenous CB5 (*cyb5* strain, A and B), CBR (*cbr1* strain, C and D), or CPR (*ncp1 *strain, E and F). Multigenes were coexpressed under the galactose-inducible promoter as indicated. *Trans*-cinnamic acid or coniferyl alcohol was fed as substrate for C4H or F5H1, respectively. After a 0.5-hour (C4H) or 3-hour (F5H1) incubation at 28°C, the ethyl acetate–extracted products were monitored by UHPLC-MS. The fold change shown on the top of the bars or solid lines indicates the change of catalytic efficiency compared with the control cells harboring C4H (A, C, and E) or F5H1 (B, D, and F) with 2muCB5D or between the solid line–indicted pairs. (**G** and **H**) In vitro enzymatic assays for C4H (G) or F5H1 (H) activity using microsomal proteins prepared from the engineered *ncp1* yeast harboring C4H or F5H1 and different electron transfer elements. *Trans*-cinnamic acid (G) and coniferaldehyde (H) were used as substrates for C4H and F5H1, respectively. NADH or NADPH was added as reductant. After 1-hour incubation at 28°C, ethyl acetate extracts were monitored by UHPLC-MS. Experiments were performed three times independently. Data are presented as means ± SD of three biological replicates from one set of representative experiment. The fold changes were shown to compare the catalytic efficiency of different constructs.

When C4H activity was monitored, the yeast cells harboring C4H with 2muCB5D exhibited a low but detectable activity that converts the fed *t*-cinnamic acid to *p*-coumaric acid, which presumably represents the background activity and was achieved with the yeast endogenous redox partners, such as the residual CPR (Fig. 8, A, C, and E). Coexpression of C4H with AtCB5D and CBR1 singly or in combination in all three yeast genetic backgrounds did not substantially alter the catalytic efficiency of C4H, compared to that coexpressed with 2muCB5D (Fig. 8, A, C, and E). However, when either ATR1 or ATR2 was coexpressed with C4H, its activity was drastically enhanced (up to 12-fold in yeast *ncp1* background), regardless of the presence or absence of AtCB5D (Fig. 8, A, C, and E). These results confirm that ATR alone is sufficient for C4H-catalyzed *para*-hydroxylation reaction and CB5 (and/or CBR) is not a necessary electron shuttle component for its catalysis.

By contrast, coexpression of F5H1 with AtCB5D substantially increased the catalytic activity of F5H in converting coniferyl alcohol to 5-hydroxconiferyl alcohol, compared to that of F5H1 coexpressed with 2muCB5D in all three yeast genetic backgrounds (Fig. 8, B, D, and F), confirming the functional association of the electron donor CB5D with F5H1. The enhancement of F5H activity with incorporation of AtCB5D alone in yeast cells probably was due to its coupling with the yeast endogenous reductase systems. Introducing AtCBR1 alone (i.e., the cells harboring CBR1, 2muCB5D, and F5H1) did not enhance F5H1 activity, confirming that CBR itself does not directly transfer electrons to P450 enzyme (Fig. 8, B, D, and F). Coupling CBR1 with AtCB5D slightly increased F5H1 activity, compared with the cells coexpressing F5H1 with AtCB5D alone (Fig. 8, B, D, and F); this minor enhancement with incorporation of CBR1 is likely due to the complementation of the yeast endogenous redox component (CPR or CBR) that is able to reduce AtCB5D. Coexpression of F5H1 with ATR1 or ATR2 alone (i.e., together with 2muCB5D) in three yeast cell genetic backgrounds, particularly in the cells deficient in endogenous CB5, only slightly enhanced F5H1 activity, compared with the yeast cells harboring F5H1 and 2muCB5D, suggesting that ATR alone has limited ability directly augmenting F5H catalysis (Fig. 8B). When F5H1 coexpressed with both ATR1/ATR2 and AtCB5D, however, its activity was substantially enhanced; this is particularly obvious in the *ncp1* yeast strain partially deficient in its endogenous CPR, showing about five times increase compared to that in the cells coexpressing F5H1 with AtCB5D and about 24-fold enhancement relative to the cells expressing F5H1 alone (Fig. 8F). These results strongly evidence that F5H1-catalyzed benzene ring 5-hydroxylation necessarily requires AtCB5D-mediated electron transfer chains, and both ATR-CB5D and CBR1-CB5D support F5H1-catalyzed hydroxylation reaction.

NADH and NADPH are the stringent reductant cofactors for *Arabidopsis* CBR and CPR (ATR), respectively (*15*). To evaluate the roles of NADH and NADPH in supporting monolignol biosynthetic P450s, microsomes were prepared from yeast *ncp1* strain harboring C4H and F5H1 and the reconstituted electron transfer pathway composed of CBR1 or ATR2, and CB5D and then used for in vitro catalytic assay. When NADH was used as the cofactor, none or negligible C4H activity was detected with all microsomal preparations (Fig. 8G), which is sharply in contrast to the high activity detected when NADPH was used as the reductant (Fig. 8G). In the presence of NADPH, microsomes harboring CBR1 alone or with CB5D yielded a basial C4H activity, probably reflects the effect of yeast endogenous residual CPR activity. The high C4H catalytic activity was only monitored using microsomes containing C4H and ATR2; moreover, the measured activity was essentially the same with the microsomes harboring ATR2-CB5D or ATR2-2muCB5D (control) (Fig. 8G), further confirming that the electron transfer from NADPH to C4H does not need the involvement of CB5 protein.

The F5H1 activity, however, could be detected with microsomes containing either CBR1-CB5D or ATR2-CB5D in the presence of either NADH or NADPH, albeit the measurable 5-hydroxylation product was less when NADH was used as the reducing power (Fig. 8H). The microsomes coexpressing F5H1 with ATR2-CB5D showed the highest F5H1 activity with NADPH as the cofactor (Fig. 8H), suggesting that NADPH-ATR2-CB5D is a preferred electron deliver pathway for supporting F5H activity. Moreover, without functional CB5D in microsomes, the F5H activity was notably depleted either in the presence of NADPH or NADH (Fig. 8H), further demonstrating the necessity of CB5D component in both NADH-CBR and NADPH-CPR pathways. In the presence of NADH, the highest F5H1 activity was observed in the yeast microsome coexpressing F5H1 with CBR1-CB5D chain (Fig. 8H), confirming that NADH-CBR1-CB5D electron pathway augments F5H1-catalyzed reaction. These in vitro assays demonstrate that both NADPH and NADH can serve as the cofactors for F5H1 catalysis, and CB5D plays crucial role in both NADPH- and NADH-related electron transfer systems for supporting the F5H activity.

## DISCUSSION

Redox partner(s) and related electron transfer chains are the indispensable part of microsomal cytochrome P450 system necessary for monooxygenase-catalyzed oxidation reaction (*3*). While the importance of P450 monooxygenases in plant chemodiversity has been well recognized and documented, the metabolic roles of redox patterns and the related electron supply chains of microsomal P450 system in various plant metabolisms are poorly investigated. Conventionally, NADPH-dependent CPR is regarded as the typical redox partner for cytochrome P450 enzymes (*10*). It delivers reducing equivalents from cofactor NADPH directly to prosthetic center of P450 enzyme in most cases; while CBR routinely couples with CB5s transferring electrons to the heme- or nonheme-containing enzymes for plant fatty acid hydroxylation, desaturation, and elongation (*13*, *19*). Our previous study, however, found that *Arabidopsis* CB5 family member AtCB5D acts as an indispensable electron donor for F5H1-catalyzed S-lignin formation (*8*). However, it is unclear which electron supply chain(s) this CB5 protein might associate with and how the related electron transfer system(s) functions in phenylpropanoid biosynthesis. Studies with the mammalian microsomal electron transfer system support that CB5, which is usually reduced by NADH-dependent CBR, can also be reduced by NADPH-dependent CPR (*5*, *6*). The reduced CB5 provides the second electron for a set of cytochrome P450 monooxygenases in drug metabolism (*6*). With the expansion of redox components in plants, i.e., two ER membrane–bound NADPH-dependent CPRs (*10*), two CBRs (*13*), and five CB5s (*20*) in *Arabidopsis*, it is possible that multiple electron transport chains can be constituted to drive P450 catalysis. This evolutionary invention, in addition to the massive recruitment of cytochrome P450 enzymes in plant kingdom, might offer greater metabolic versatility to yield the amazing repertoires of diverse plant-specialized metabolites.

**Fig. 9.**
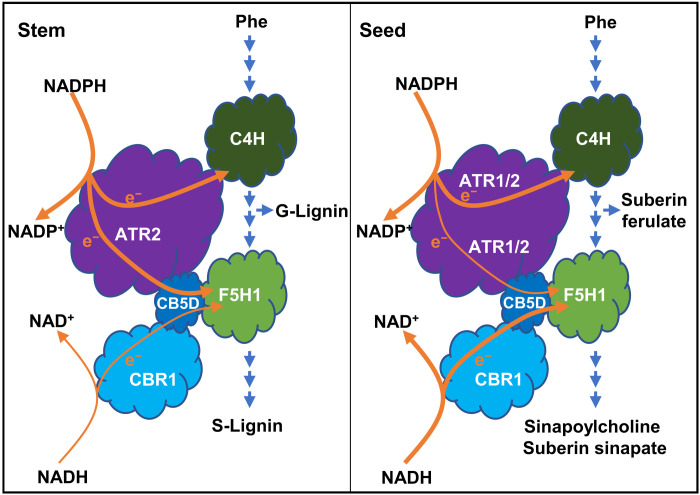
The proposed model of differential recruitment of electron transfer pathways for C4H- and F5H1-catalyzed phenolic syntheses in *Arabidopsis* stem and seed. C4H uses NADPH-dependent ATR electron transfer chain to deliver reducing power from reductant NADPH. While ATR2 acts as the prime redox partner in stem to directly deliver electrons to C4H for both G- and S-lignin synthesis, ATR1 and ATR2 appear to redundantly function for seed coat ferulate formation. On the other hand, both NADH and NADPH are the electron donors for F5H1-catalyzed reactions; CB5D acts as an electron shuttle hub delivering electrons from NADPH-ATR and NADH-CBR pathways to F5H1. In stem, NADPH-ATR2-CB5 electron transfer pathway functions dominantly for F5H1-catalyzed S-lignin biosynthesis; whereas, in seed, the NADH-CBR1-CB5 electron transfer pathway plays primary roles in supplying reducing power to F5H1 for sinapoylcholine and suberin sinapate syntheses.

Aligning with such perspectives, in the present study, we discover that phenylpropanoid-monolignol biosynthetic P450 systems recruit distinct redox partners/electron transfer chains to sustain their catalysis for 4- and 5-hydroxylation of benzene ring. As depicted in Fig. 9 of the conceptual working model, evidences from our whole-cell biocatalytic assay, in vitro microsomal enzymatic activity determination and in planta genetic characterization corroborate that the evolutionarily early emerged C4H in the general phenylpropanoid pathway (*31*, *32*), as the most of conventional P450 enzymes, couples with NADPH-CPR electron transfer chain for its catalysis to produce *para*-hydroxylated phenolics, in which CPR directly reduces C4H without requirement of additional electron shuttles such as CB5 (Fig. 9). Coupling NADPH-ATR-CB5 or NADH-CBR-CB5 electron chain showed no influence on C4H activity (Fig. 8). Consistently, the loss of *CBR1* or *CB5* in planta exhibited no impairment on stem G-lignin deposition and seed coat ferulate synthesis (Figs. 4A and 6B). Disruption of *AtCB5D* alone or all microsomal *CB5* isogenes even enhanced stem G-lignin content or suberin ferulate in seed coat (Figs. 1D, 4B, and 6B), which probably reflects the redirection of carbon flux to the guaiacyl derivatives along with the suppression of F5H-mediated 5-hydroxylation of phenolics. By stark contrast, the F5H-catalyzed benzene ring 5-hydroxylation necessarily requires CB5 proteins. Disruption of microsomal *CB5* genes resulted in ~70% (or more) reduction of S-lignin monomer in stem, sinapoyl malate in leaves, sinapoylcholine in seeds, and suberin sinapate of seed coats (Figs. 1, C and D, 5B, and 6C), which are consistent with our previous finding that AtCB5D is an indispensable electron donor for F5H-catalyzed reaction (*8*). Considering the nearly complete loss of S-lignin synthesis when disruption of F5H1 (*28*), the data herein suggest that *Arabidopsis* CB5s support the majority of phenyl ring 5-hydrxoyaltion activity of F5H1 and thus play prime role in supplying electrons to this P450. Similar to the mammalian CB5, AtCB5D can be effectively reduced by either NADPH-CPR (ATR2) or NADH-CBR1 at the catalytic concentration of either reductase (Fig. 3). When coexpressed in yeast cells, both NADPH-ATR2-CB5D and NADH-CBR1-CB5D enhanced F5H catalytic activity (Fig. 8). Consistently, disruption of either *CBR1* or *ATR2* (and/or *ATR1*) caused the notable depletion in S-lignin synthesis or the formation of 5-hydroxyalted phenolic esters in seeds and seed coats (Figs. 4 to 6). These data strongly suggest that F5H uses both NADPH-ATR-CB5 and NADH-CBR-CB5 electron transfer pathways for its catalysis, in which CB5D functions as an electron shuttle hub that accepts electrons from both supply chains to support F5H catalysis (Fig. 9). This unique feature confers great catalytic versatility to the latest evolved P450 F5H1 for its 5-hydroxyaltion of phenolics in higher plants.

More intriguingly, our study reveals that different electron supply chains preferentially associate with F5H1 in different tissues for synthesizing different classes of 5-hydroxylated phenolics (Fig. 9). Analyzing *Arabidopsis* mutants deficient in redox components clearly shows that NADPH-ATR2-CB5D electron chain plays a dominant role in supporting F5H activity for stem S-lignin synthesis (Fig. 4). In contrast, disruption of *CBR1* yielded much severer depletion on seed soluble and seed coat sinapate than did the loss of *ATR1* or *ATR2* (Figs. 5 and 6), suggesting that the NADH-CBR1-CB5 route takes the prime role in the F5H-catalyzed seed and seed coat sinapate esters synthesis. While the conventional NADH-CBR1-CB5 pathway dominates seed sinapoyl ester biosynthesis (Figs. 5 and 6), none of the single reductase (CBR1, ATR1, or ATR2) after its disruption affected leaf sinapoyl malate accumulation, while the loss of CB5 strongly comprised its synthesis (fig. S9). These results implicate that the reductases existing in the leaf tissue might play overlapped functions that associate with AtCB5D to cooperatively transfer reducing equivalent to F5H for leaf sinapoyl ester synthesis. Sinapoylcholine is found at high concentrations in the seeds of the agronomically important crop *B. napus* (oilseed rape) (*43*). Because of its antinutritive property, engineering sinapoylcholine metabolism to reduce its biosynthesis in the seeds of *B. napus* could improve the nutritive quality of canola meal (*27*). On the other hand, the leaf sinapoyl malate is involved in protecting plants against the deleterious UV-B radiation. Discovery of the differential requirements of electron transfer systems in respect to the synthesis of different classes of 5-hydroxylated phenolic derivatives in leaf, stem, and seed offers powerful tools to manipulate phenolic synthesis in a tissue-specific manner.

The molecular mechanisms governing tissue-preferential recruitment of microsomal electron transfer chains to synthesis of different classes of tissue-specific phenolics remain to be further explored. Nonetheless, evidence from the present study suggest that plant might use multiple means to achieve such a functional differentiation of electron transfer chains. First, the transcriptional regulation is imposed on the genes encoding redox components. *ATR1*, *ATR2*, and *CBR1* show perceivable tissue-preferential expression patterns in the examined leaf, stem, flower, and silique tissues; the differences in transcript abundance might define the levels and activities of those reductases in different tissues (Fig. 7). Second, the availability of pyridine nucleotide cofactors NADPH and NADH might serve another layer of regulatory mechanism that determines which electron transfer chain(s) being recruited in phenolic synthesis. Studies on mammalian and plant CBR and CPR enzymes have demonstrated their stringent specificity to the cofactors NADH and NADPH, respectively (*15*, *44*). Although the yeast microsome containing F5H1 and CBR1 or F5H1 with CBR1-CB5D exhibited considerable 5-hydroxylation activity in the presence of NADPH (Fig 8H), those activities most likely were gained from the complementation of yeast endogenous CPR and/or its association with AtCB5D, because the yeast strain *ncp1* used in the experiments is the heterozygous mutation of CPR (*42*). While both NDAH and NADPH cofactors are abundantly presented in the developing seeds, the level of the former is drastically low in the developing stem (fig. S14). Although the redox status in plant tissues might be dynamic and fluctuate in different types of cells or even in different subcellular compartments, the constant low abundance of NAD(H) in the stem tissue offers a potential explanation on the observed limited contribution of NADH-CBR1-CB5 electron transfer pathway to the F5H-catalyzed S-lignin synthesis in stem, even though *CBR1* and *AtCB5D* transcripts are abundantly detected in the developing stem tissue. On the other hand, ATR and CBR1 display differential reductase properties toward different electron acceptors. When AtCB5D as the acceptor substrate, the amount of CBR1 required to completely reduce CB5 is approximately 60 times lower than that of ATR2 (Fig 3), suggesting more efficient electron transfer of NADH-CBR1 to CB5 protein than that of NADPH-ATR. Together with the limited expression of *ATR* genes in developing seeds, this explains the reason that CBR-CB5 electron transfer pathway becomes the dominant player in supporting F5H1-catalyzed 5-hydroxylation of phenolics in *Arabidopsis* developing seeds where both NADH and NADPH are abundantly available (fig. S14). This interpretation however should not be confused with the observation that NADPH-ATR-CB5 pathway is more effective in supporting F5H activity in yeast heterologous system, because where ATR is substantially overproduced.

In yeast and mammalian systems, the CB5-mediated electron transfer chains (NADH-CBR-CB5 and NADPH-CPR-CB5) support a number of P450 activities (*6*). In plant, the evidence of CB5 as electron donor for P450-catalyzed reactions is emerging (*45*). Besides the described NADH-CBR-CB5 and NADPH-CPR-CB5 electron transfer routes for *Arabidopsis* F5H1 (CYP84A1) in the present study, an early investigation also demonstrated that disruption of a single *Petunia hybrida cb_5_* locus, *DifF*, resulted in discoloration of flower petals and compromised the activity of flavonoid 3′,5′-hydroxylase (F3′5′H; CYP75A) involved in the biosynthesis of delphinidin-based anthocyanins (*9*), which implicates that CB5 and the related electron transfer chain(s) are required for F3′5′H-catalyzed reaction in flavonoid biosynthesis. In addition, our previous study also revealed that *Arabidopsis* CB5D is an indispensable electron donor for AtF5H2 (CYP84A4), the AtF5H1 close paralog that catalyzes the formation of α-pyrones (*46*). In *cb5d* mutant, the contents of the major α-pyrones arabidopyl alcohol and iso-arabidopic acid were reduced by 80% compared to the WT (*8*). However, disruption of *CB5D* did not affect *Arabidopsis* F3′H (CYP75B)–catalyzed accumulation of flavonols (*8*). AtF3′H is an evolutionarily close homolog of AtF5H1 and AtF5H2 for synthesis of cyanidin-based anthocyaninins (*24*). These data suggest that CB5 as an electron donor, differing from CPR, likely has relatively strict functional specificity and supports a particular set of P450 enzymes in planta. F5H1 as a recently evolved P450 enzyme distributes widely in angiosperms for 5-hydroxylated phenolic synthesis. It is interesting to further explore whether the catalysis of F5H homologs in other angiosperm species is also CB5 dependent. S-lignin has also been detected in the basial lineage of vascular plant lycophytes. An independently evolved cytochrome P450 monooxygenase in lycophyte *Selaginella moellendorffii*, denoted as SmF5H, is capable of converting guaiacyl intermediates to S-lignin monomer (*24*). It is interesting to further determine whether CB5-mediated electron delivery pathway(s) is needed for SmF5H in lower vascular plant.

Besides the involvement of CB5 in the P450 systems for phenolic formation, sporadic evidence also suggest that CB5-mediated electron transfer chains might be involved in other specialized metabolisms. Glucosinolates are nitrogen- and sulfur-containing small-molecule compounds found in the order *Brassicales*. Disruption of *AtCB5C* did not alter the overall glucosinolate biosynthesis but influenced the accumulation of particular long-chain aliphatic glucosinolate species, especially when induced by methyl jasmonate treatment (*34*). This infers a potential functional association of CB5 with CYP79F that catalyzes the key step in the biosynthesis of long-chain aliphatic glucosinolates. In addition, in the effort of metabolic engineering of artemisinic acid biosynthesis, expression of *Artemisia annua* CYP71AV1 with its cognate reductase CPR1 in yeast (*S. cerevisiae*) rendered severe oxidative stress due to the poor coupling of cytochrome P450 and reductase thus releasing reactive oxygen species (*47*). Integration of a *A. annua* CB5, together with other catalytic enzymes, greatly improved artemisinic acid yield (*47*), implicating that CB5-mediated electron transfer might be necessary for *A. annua* CYP71AV1 activity.

Our genetic and biochemical data clearly show that CB5 is an obligate electron donor supporting AtF5H1 but not AtC4H activity. The underlying mechanism governing such functional specificity remains unresolved. In mammalian system, it is well determined that CB5 binds to the basic proximal surface of P450 via electrostatic interactions to deliver electron to the prosthetic center of P450 (*16*). Therefore, one of the possibilities is that the recently evolved AtF5H1 (CYP84A) has distinct structural features, which favors for the interaction with CB5 redox partner. Nevertheless, our immunoprecipitation–mass spectrometry analysis, bimolecular fluorescence complementation assay, and even yeast two-hybrid test revealed that AtCB5D appears to physically interact with C4H and C3′H besides F5H (*8*). It has been proposed that electron transfer proteins follow a two-step model for complex formation: first, the formation of an ensemble of encounter complex, in which the two partners adopt different orientations relative to one another, and then the establishment of a well-defined stereospecific complex, in which electron transfer occurs. The long-range, nonspecific electrostatic interactions drive the encounter complex formation, while the specific, short-range interactions (e.g., hydrogen bonds, salt bridges, and hydrophobic interactions) hold the stereospecific complex(es) (*48*). Therefore, presumably some unique structural features of F5H enable it to interact with CB5 to form electron transferring stereospecific complex, while the interactions of CB5 with C4H or C3′H only form the nonspecific encounter complex. Cryo–electron microscopy–mediated single-particle structure determination of CB5-P450 complex(es) might be helpful to elucidate this intriguing issue.

Although NADH-CBR1 and NADPH-ATR electron transfer chains exhibit partially redundant functions in transferring reducing power to F5H, the terminal electron donor CB5 proteins show high specificity in supporting F5H-catalyzed reaction. Except for AtCB5D, none of the other microsomal CB5 members effectively complements the defects of *cb5d-1* mutant in the 5-hydroxyalted phenolic accumulation (Fig 1, E and F). Simultaneously, disrupting all microsomal CB5 members resulted in none or only a limited additive effect on S-lignin monomer and sinapoyl ester syntheses, compared with the *AtCB5D* single mutant (Figs. 1, A to D, and 5B). Such genetic evidence strongly suggests that CB5 family members have functional specialization in planta. Sporadic evidence suggest that AtCB5B might be part of an alkane-forming complex. Its physical interaction with ECERIFERUM1 (CER1) or CER1-like protein presumably mediates electron transfer to the catalytic site of the later thus participating in *n*-alkane biosynthesis (*21*, *49*). In addition, characterization of AtCB5C T-DNA insertion mutant alleles suggests its potential involvement in the biosynthesis of glucosinolate species (*34*). Given the high sequence similarity and structural conservation of CB5 family members (*8*, *18*, *20*), it will be interesting to further explore how they discriminate themselves for different biological functions.

Together, the present study discovers additional layer of metabolic complexity that the higher plants have evolved, in which the ancillary redox components of P450 system are selectively recruited in different tissues to control different classes of phenolic synthesis. Understanding the functional differentiation of electron transfer components in phenylpropanoid-monolignol biosynthetic P450 systems in different tissues is biotechnologically meaningful toward precisely and differentially tailoring phenolic profiles for optimizing plant feedstocks.

## MATERIALS AND METHODS

### Plant materials and growth conditions

The *A. thaliana* mutant lines *cb5a-1* (SALKseq_054962), *cb5b-1* (SALK_100161), *cb5c-1* (SALK_027748), *cb5d-1* (SALK_045010), *cb5d-2* (GABI_328H06), *cb5e-1* (SALK_151509), *atr1-1* (SALK_208483), *atr1-2* (SALKseq_051672), *atr2-1* (SALK_152766), and *fah1-2* were ordered from the Arabidopsis Biological Resource Center. Homozygous T-DNA insertion mutants were obtained by genotyping with genomic DNA as template. *cb5c-cr* mutants were generated by the CRISPR-Cas9 method following described procedure (*50*). To generate high-order mutants, single or double mutants were crossed to obtain F1 generation seeds. Double and quadruple mutants were obtained from F2 progeny by PCR genotyping or sequencing. The primers used for genotyping are listed in table S1. For seed germination, seeds were sterilized with 70% alcohol and sown on half Murashige and Skoog medium (Phytotech, M524) containing 1% agar and 1% sucrose. After 3-day stratification at 4°C, the seeds were germinated and maintained at 22°C under a 16-hour light/8-hour dark regime in a growth chamber (BioChambers) with a light intensity at 8500 lumens/m^2^. The 7-day-old seedlings were transferred to soil and grew until mature under the same conditions.

### qPCR analysis of gene expression

Total RNAs were extracted from *Arabidopsis* materials in triplicate using TRIzol reagent (Thermo Fisher Scientific) following the manufacturer’s instruction. Reverse transcription reaction was conducted using 1 μg of total RNA and 4 μl of iScript Reverse Transcription Supermix (Bio-Rad) in a 20-μl reaction volume. The complete reaction mix was incubated in the thermal cycler at 25°C for 5 min, 46°C for 20 min, and 95°C for 1 min. The cDNA solution was diluted five times, and 2 μl of the diluted solution was used as template in a 15-μl reaction with SsoAdvanced Universal SYBR Green Supermix (Bio-Rad). qPCR was performed using the CFX96 Real-Time System (Bio-Rad), and the cycle threshold value was calculated using the CFX Manager Software v.3.3 (Bio-Rad). Primers used in qPCR are listed in table S1. *Arabidopsis PP2A* or *EF1*α*A4* gene was used as the housekeeping reference gene, and the data were calculated using the delta-cycle or delta-delta–cycle threshold method (*51*).

### Plant transformation

For *cb5d-1* complementation test, the coding regions of *CB5* genes were amplified by PCR with the gene specific primers (table S1). All the genes were cloned to the gateway cloning entry vector pDONR207 (Thermo Fisher Scientific) and sequenced, thereby creating entry clones. The CB5 genes in entry vectors were subcloned to a destination vector pMDC32-pC4H that was generated previously (*52*), in which *Arabidopsis C4H* promoter was used to drive the target gene expression. The obtained constructs were transferred into Agrobacterium strain GV3101. Then, the constructs were transformed into *cb5d-1* mutants using the floral dip method (*53*). The primary transformants were screened on ½ MS plates containing hygromycin B (15 μg ml^−1^; Gold Biotechnology). After 2 weeks, the positive seedlings were transferred to soil and grew to mature in a growth chamber with the above-described conditions.

### Soluble phenolic quantification

The leaf soluble phenolics were analyzed following the described method (*52*) with some modifications. Briefly, two 4-week-old rosette leaves from different plants were sampled and weighed into a 1.5-ml centrifuge tube. Three hundred microliters of 80% (v/v) methanol containing 80 μM chrysin (as internal standard) and two steel balls were added to each tube. Leaves were ball-milled using a CryoMill (Retsch) for 2 min at 30 Hz. Then, additional 200 μl of 80% (v/v) methanol containing 80 μM chrysin buffer was added to each sample. After 4-hour extraction at 4°C, 5 μl of the supernatant was injected to a UHPLC hyphenated with a Q Exactive plus MS system (Thermo Fisher Scientific) and resolved with a reverse phase C18 column (Luna, 150 mm by 2.1 mm, 1.6 μm; Phenomenex) using a gradient program: 5% B (0 min), 30% B (1 min), 80% B (19 min), 99% B (20 min), holding for 2 min, and then to 5% B (23 min) at a flow rate of 0.1 ml/min. Mobile phases A and B were 0.1% acetic acid aqueous solution and 0.1% acetic acid in acetonitrile. The compounds were detected with ultraviolet-visible (UV-Vis) diode array detector at 265, 280, 330, and 510 nm wavelengths. The mass spectrometer parameters were set as the following: negative model; sheath gas flow rate, 40 U; auxilary (aux) gas unit flow rate, 10; capillary temperature, 350°C; aux gas heater temperature, 250°C; spray voltage, 3.5 kV; S lens Re-Imagined Focus (RF) level, 60. Thermo Xcalibur 4.1 software was used to process the data. For quantification of the detected sinapoyl esters, including sinapoyl malate and sinapoylglucose, the absorbance peak area at 330 nm was normalized with that of the internal standard chrysin and with the respective sample fresh weight. The relative content was expressed by comparing with that of the WT.

Seed-soluble phenolic analysis was performed following procedure described previously (*26*). Briefly, 10 mg of dry seeds was mixed with 300 μl of 50% methanol containing 1.5% acetic acid, and 100 μM chrysin as internal standard was added and ball-milled using a CryoMill (Retsch) for 2 min at 30 Hz. Nine hundred microliters of 50% methanol containing 1.5% acetic acid and 100 μM chrysin was then added to each sample. The samples were rotated in cold room for 1 hour before centrifugation at 20,000*g* (Eppendorf 5424R centrifuge; FA-45-24-11 rotor) twice. Then, identification of seed-free phenolics was performed. One microliter of extracted aliquot was injected into the above-described UHPLC-MS system and resolved using a gradient program of mobile phase A in 5% B (0 min), 20% B (1 min), 30% B (19 min), 99% B (20 min), 99% B (27 min), and 5% B (28 min) at a flow rate 0.1 ml/min. A and B were 0.1% acetic acid aqueous solution and 0.1% acetic acid in acetonitrile. The compounds were detected with UV-Vis diode array detector at 330-nm wavelengths and a mass spectrometer. For the detection of sinapoylcholine, the mass spectrometer parameters were set as the following: positive model; sheath gas flow rate, 40 U; aux gas unit flow rate, 8; capillary temperature, 350°C; aux gas heater temperature, 250°C; spray voltage, 3.5 kV; S lens RF level, 50. All the other settings and the data processing steps were the same as described for leaf soluble phenolic analysis. For seed phenolic quantification, 5 μl of extracted aliquot was analyzed with an Agilent 1100 HPLC system with a gradient of solvent B (0.1% acetic acid in acetonitrile) in solvent A (0.1% acetic acid in water) as follows: 15% B (0 min, 0.8 ml/min), 15% B (2 min, 0.8 ml/min), 30% B (25 min, 0.8 ml/min), 100% B (30 min, 1 ml/min), 100% B (35 min, 1 ml/min), and 15% B (36 min, 0.8 ml/min) in a C18 column [Luna C18 (2), 5 mm; Phenomenex]. The detection wavelengths were 265, 280, 310, 330, and 510 nm. To quantify the contents of seed-soluble phenolics, the UV absorption peak area at 330 nm of both sinapoylcholine and sinapoylglucose from each sample was normalized to that of the internal standard (chrysin) and calibrated using a standard curve of sinapic acid.

### Lignin composition analysis

For the stem lignin composition analysis on the developmental plants, the full stems were sampled at the development stage from 5th to 11th week after they were planted in the soil. Otherwise, for the other experiments, the 10-cm basal stems were harvested from 11-week-old *Arabidopsis* plants. The stem samples were lyophilized and then ball-milled using a CryoMill (Retsch, Haan) for 2 min at 30 Hz. The obtained powder was extracted with 70% ethanol at 65°C for 3 hours. The step was repeated three times. Then, the residues were extracted with chloroform/methanol (1:1, v/v) for 1 hour, repeated three times. After one-time overnight acetone extraction, the residues were dried at room temperature to obtain extractive-free cell wall residues. The lignin monomeric composition was analyzed with a thioacidolysis method following the described procedure (*54*) with minor modifications. Briefly, about 10 mg of extractive-free residues was weighed into a glass vial. A freshly prepared reaction mixture (2.5% boron trifluoride etherate and 10% ethanethiol in dioxane, v/v) was added to each sample. The vials blew with nitrogen gas before sealing. The vials were placed in 95°C heat block for 4 hours with periodic agitation. After cooling down, 0.3 ml of sodium bicarbonate (0.4 M), 2 ml of water, and 1 ml of methylene chloride [containing tetracosane (1 mg/ml)] were added successively. The vials were vortexed for 1 min and phase-separated by centrifugation. About 1 ml of the organic phase was collected and dried in a heat block (50°C) overnight. Methylene chloride (0.5 ml) was added to resuspend the dried sample, and 50 μl of the sample was transferred to a new centrifuge tube and dried under the same condition. Derivation was performed by adding 50 μl of pyridine and 50 μl of *N*-methyl-*N*-(trimethylsilyl) trifluoroacetamide and incubation at room temperature for 5 hours. One microliter of product was injected to an Agilent 7890A gas chromatograph with a flame ionization detector and resolved with an HP-5MS fused silica capillary column (30 m by 0.25 mm, 0.25-μm film thickness; Agilent). The temperature of inlet and detector was held at 250° and 300°C, respectively. The column flow was set to 1.5 ml/min. The oven temperature was programmed from 130°C, ramped to 180°C at 10°C/min, then to 255°C at 3°C/min, and held for 5 min. Lignin monomer content was quantified as described before with a response factor of 1.5, defined as the ratio of relative concentration to relative area, using tetracosane as the internal standard (*55*). Lignin S/G ratio was calculated by dividing the peak areas of S-lignin monomer derivatives by G-lignin monomer derivatives.

### Acetyl bromide total lignin content analysis

Total lignin content was measured by acetyl bromide method (*56*). Briefly, 1 mg of extractive-free cell wall residues was incubated with 100 μl of freshly prepared acetyl bromide solution [25% (v/v) acetyl bromide in glacial acetic acid] at 50°C for 2 hours. Then, an additional hour of incubation with vortexing every 15 min was performed. After cooling to room temperature, 400 μl of 2 M sodium hydroxide and 70 μl of freshly prepared 0.5 M hydroxylamine hydrochloride were added to the reaction. Glacial acetic acid (1430 μl) was added to bring up the solution volume to 2 ml. Two hundred microliters of solution was pipetted into a UV-specific 96-well plate (Corning, Kennebunk), and the absorbance at 280 nm was measured in a Spark microplate reader (Tecan, Männedorf). The extinction coefficient of 15.69 g^−1^ cm^−1^ was applied for the calculation of lignin content.

### Mating-based split-ubiquitin yeast two-hybrid assay

The mating-based split-ubiquitin yeast two-hybrid system was adopted to test the interactions of electron transfer components (*57*). The coding sequences of *CB5*, *ATR1*, *ATR2*, and *CBR1* gene were amplified by PCR with the gene-specific primers (table S1). All the genes were cloned to the pDONR207 vector (Thermo Fisher Scientific), thereby creating entry clones. Then, LR reaction was performed to generate pMetYC-Gene1 bait constructs (i.e., ATR1, ATR2, and CBR1) and pNX32 -Gene2 prey constructs (i.e., CB5A to CB5E). The bait and prey vectors were transformed to THY.AP4 and THY.AP5 yeast strains and grown on SC/-Leu and SC/-Trp plates (Takara), respectively. After 3 days of incubation at 28°C, 10 colonies of transformed yeast were inoculated in corresponding media and grown overnight. The yeast cells were harvested and mated. The mated yeast cells were selected on SC/-Trp, -Leu, and -Ura growth plates (Takara) and serially diluted to SC/-Trp, -Leu, -Ura, -Ade, -His, and -Met selection plates (Sunrise Science Products) and incubated at 28°C. Images were photographed after 3 to 5 days of growth.

### Protein expression, purification, and reductase assay

The CB5D, ATR1, and ATR2 coding sequences without the transmembrane domain were PCR-amplified and inserted to pET28a (+) vector via Eco RI and Xho I restriction enzyme sites. The CBR1 coding sequence without the transmembrane domain was cloned to pMAL-c2X vector using Eco RI and Sal I enzyme sites. Then, the MBP-CBR1 fusion sequence was PCR-amplified and religated to pET28a (+) via Eco RI and Xho I sites. The obtained constructs were transformed into *Escherichia coli* BL21 cells for protein expression. Twenty milliliters of overnight cultures was inoculated to 600 ml of Terrific broth and grew at 37°C till an optical density of around 1 at 600 nm and then grew at 15°C for 30 min before induction with 0.5 mM isopropyl-β-d-thiogalactopyranoside. Overnight cultures were pelleted and resuspended with lysis buffer [20 mM tris-HCl (pH7.5) containing 200 mM NaCl, 10 mM imidazole, 0.1% Triton X-100, 1 mM phenylmethylsulfonyl fluoride (PMSF), 5 mM β-mercaptoethanol, and 1× cocktail protease inhibitors], sonicated in an ice bath, and spined at 9682*g* (Sorvall RC 5C Plus centrifuge, SS-34 rotor) for 1 hour. The cleared lysate of CBR1, ATR1, and ATR2 was incubated with Ni^2+^–nitrilotriacetic acid agarose beads for 1 hour at 4°C. For purification of CB5D proteins, the supernatants were incubated with 100 μM hemin for 1 hour at 4°C before incubating with Ni^2+^–nitrilotriacetic acid agarose beads. After transferring to a gravity flow column and draining the liquid, the beads were washed with washing buffer [20 mM tris-HCl (pH7.5) containing 200 mM NaCl, 20 mM imidazole, 1 mM PMSF, and 5 mM β-mercaptoethanol] till no protein was eluted. The recombinant proteins were eluted with elution buffer [20 mM tris-HCl (pH7.5) containing 200 mM NaCl and 300 mM imidazole] and then desalted to 20 mM tris-HCl buffer (pH7.5) containing 200 mM NaCl using the Bio-Gel P-6DG gel following the manufacturer’s instruction (Bio-Rad). CBR1, ATR1, and ATR2 protein concentration was quantified with Bradford Reagent (B6916, Sigma-Aldrich). CB5D protein concentration was quantified from the different spectra of the cytochrome catalyzed by dithionite using the extinction coefficient of ∆ε(reduced-oxidized)_424–409_ = 185 mM^−1^ cm^−1^ or from the absolute spectrum using extinction coefficient ε_413_ = 117 mM^−1^ cm^−1^ (*58*). The purified protein was aliquoted and stored at −80°C for further use.

The redox assay was performed in 100 μl of 20 mM tris-HCl buffer (pH7.5), with 100 μM NADH or NADPH in a 96-well microplate, with either CB5D or Cyt C as electron acceptors. After a 5-min incubation at room temperature of the electron acceptors (CB5D or Cyt C) and electron donors (CBR1, ATR1, or ATR2), the reduction of CB5 or Cyt C is started by addition of NADH or NADPH, and then the absorbances were recorded every 10 s at 424 nm for CB5 or 550 nm for Cyt C using a Spark microplate reader (Tecan, Männedorf). The CBR1 and ATR2 catalytic concentrations for CB5 and CytC were confirmed by gradient concentrations of CBR1 and ATR2, separately. Tau values were determined by nonlinear regression analysis on the data of Δ absorbance (AU) at the function of reductase concentration fit to the one-phase association equation, implemented within GraphPad Prism 4.

### Suberin aromatic analysis

Extract-free residues were prepared from the ground seeds. Depolymerization was performed following the described method (*39*) with minor modifications. Briefly, 10 mg of extract-free residues was incubated with 2 ml of boron trichloride/methanol (12%; Sigma-Aldrich) for 2 hours at 80°C. After cooling down, 100 nmol of chrysin was added as the internal standard, and 1 ml of 0.9% NaCl was added. Then, 3 ml of chloroform was used to extract the hydrolysates twice. The extracts were pooled and dried under a stream of N_2_ gas. The dried residues were dissolved in 100 μl of 80% methanol, and 1 μl of product was injected to a UHPLC-MS system with program the same as the above described for leaf phenolic analysis to identify peaks. For the phenolic quantification, 5 μl of extracted aliquot was analyzed with an Agilent 1100 HPLC system with a gradient of solvent B (0.1% acetic acid in acetonitrile) in solvent A (0.1% acetic acid in water) as follows: 35% B (2 min), 44% B (16 min), 100% B (23 min), 100% B (25 min), and 35% B (26 min) at a flow rate of 0.8 ml/min in a C18 column [Luna C18 (2), 5 mm; Phenomenex].

### Quantification of NAD(H) and NADP(H) levels

NAD(H) and NADP(H) levels in *Arabidopsis* leaves, seeds, and stems were quantified using NAD/NADH-Glo and NADP/NADPH-Glo assay kits (Promega, Madison), respectively, following the manufacturer’s instruction. Briefly, rosette leaves from 30-day-old plants, seeds collected from 9- to 12-day postanthesis siliques, and stems from 42-day-old plants were collected at series of time points of the day with 3-hour intervals and then lyophilized, ground into fine powder using CryoMill (Retsch), dissolved in phosphate-buffered saline (137 mM NaCl, 2.7 mM KCl, 10 mM Na_2_HPO_4_, and 1.8 mM KH_2_PO_4_) with a ratio of 500 μl of buffer/1 mg of dried leaves or seeds and 5 μl of buffer/1 mg of dried stem. After a 10-min incubation on ice, 200 μl of chloroform was added and vortexed to remove proteins and chloroform-soluble compounds. With 10-min centrifugation at 20,000*g* with Eppendorf 5424R centrifuge (FA-45-24-11 rotor), the aqueous extracts were proceeded to quantify NAD(H) and NADP(H). For every sample, two portions of 50-μl extracts were added to two parallel wells of a white Opaque 96-well microplate (PerkinElmer). Fifty microliters of NAD/NADH-Glo or 50 μl of NADP/NADPH-Glo detection reagents was added to the first or second portion of samples, respectively. After gentle and brief shaking, the luminescence signals were recorded using a Spark microplate reader (Tecan, Männedorf) for 30 cycles with 2-min intervals.

To quantify NAD(H) and NADP(H) contents in yeast cells, about 3.6 × 10^8^ overnight-grown yeast cells for *cyb5*, *cbr1*, and *npc1* strains in Yeast Peptone Dextrose Adenine (YPDA) medium were harvested and moved to a 2-ml screw cap tube. Glass beads (0.5 ml; 0.45 to 0.50 mm in diameter) and 0.7 ml of ammonium acetate [50 mM (pH 8) saturated with N_2_ gas] were added. Yeast cell walls were bead-blasted using a CryoMill (Retsch) for 2 min at 30 Hz (30-s on and 120-s interval). The cell lysates were recovered, and the beads were washed twice with 1 ml of acetonitrile:50 mM ammonium acetate (3:1, v/v, saturated with N_2_ gas). The obtained supernatants were pooled and further centrifuged at 20,000*g* for 5 min to remove cell debris. Two milliliters of ice-cold chloroform was added, agitated vigorously for 30 s, and phase-separated by centrifugation. The upper aqueous phase was collected and stored at −80°C. The quantification of NAD(H) and NADP(H) was performed using NAD/NADH-Glo and NADP/NADPH-Glo assay kits (Promega, Madison) as the above described.

### Yeast expression and whole-cell feeding assays of P450s

All the constructs applied in yeast expression were using *pYeDP60* vector (*59*), which was digested with Bam HI and Eco RI restriction enzymes. The coding sequences of target genes were amplified by PCR with the specific fusion primers (table S1) and inserted to *pYeDP60* vector using the Gibson assembly method (*60*). Constructs were transformed into the yeast strain deficient in its endogenous *CB5* (*cyb5*, MAT-a, Transomic), *CBR1* (*cbr1*, MAT-a, Transomic), and *ATR* (*ncp1*, heterozygous diploids, Transomic), and positive colonies are selected by auxotrophic yeast medium devoid of uracil. The fresh yeast transformants were inoculated in 2 ml of selective medium containing 2% glucose and grow overnight at 250 rpm, 28°C. Overnight cultures (0.5 ml) were diluted into a 15-ml selective medium containing 2% glucose and incubated at 28°C, 250 rpm. After about 6 hours of incubation, the yeast cells were harvested and resuspended to an optical density at 600 nm value of 0.2 with a selective medium containing 2% galactose to induce gene expression. After about 14 hours of induction, yeast cells were harvested and washed once with TE buffer [10 mM tris-HCl (pH 7.4) and 1 mM EDTA]. For whole-cell feeding assay, yeast cells were resuspended in TE buffer to a cell density of about 1.5 × 10^8^/ml. For the assays of C4H activity, 0.5 ml of yeast cells was incubated with 250 nmol of *t*-cinnamic acid, and for F5H1 activity assay, 1 ml of yeast cells were incubated with 250 nmol of coniferyl alcohol in a 2-ml tube. Then, a 5-mm glass bead was added to each centrifuge tube to help mixing. The samples were incubated at 28°C with slow rotating for 0.5 hours (C4H) or 3 hours (F5H1). The reactions were stopped by adding 0.1 ml of glacial acetic acid and extracted with 0.5 ml of ethyl acetate containing 0.1 mM chrysin as the internal standard. The extracts were dried with a centrivap benchtop vacuum concentrator (Labconco) and resuspended with 0.1 ml of 80% methanol. One microliter of sample was injected to an UHPLC-Q Exactvie MS system (Thermo Fisher Scientific) with the same setting described as above. Peaks were identified on the basis of UV and mass spectra of the respective products. For quantification of the products, the UV absorption peak area of a particular compound from each sample was normalized to that of the internal standard (chrysin) and calibrated using a standard curve of *p*-coumaric acid or coniferyl alcohol (for 5-hydroxyconiferyl alcohol).

### Yeast microsome preparation and enzyme activity assay

After galactose induction for protein expression, the yeast cells were collected and used for microsome isolation according to the methods described (*61*). Briefly, the yeast cells were washed once with buffer A [50 mM tris-HCl (pH 7.4), 2 mM EDTA, and 100 mM KC1] and then resuspended in 1 ml of buffer B [50 mM tris-HCl (pH 7.4), 2 mM EDTA, and 0.6 M sorbitol] per 0.5 g of wet cell pellet mass; the same volume of glass beads (0.45 to 0.50 mm in diameter) was added. Yeast cell walls were disrupted mechanically by handshaking for 10 min (30 s of shaking and 30-s interval) in a cold room. The crude extracts were recovered. The beads were washed twice with an equal volume of buffer B. The three extracts were pooled together and centrifuged at 7650*g* (Sorvall RC 5C Plus centrifuge, SS-34 rotor) for 10 min twice to recover the supernatant. Polyethyleneglycol 4000 (0.1 g/ml final) and NaCl (8.8 mg/ml final) were added to the supernatant and incubated on ice for 30 min. Microsomal fractions were obtained by centrifugation at 9682*g* (Sorvall RC 5C Plus centrifuge, SS-34 rotor) for 20 min and resuspended in buffer C [50 mM sodium Pipes (pH 7.0) containing 4 mM EDTA and 20% glycerol]. Microsome enzyme activity assays were performed in buffer C in a volume of 100 μl containing 100 μg of microsomal protein, 0.2 mM substrate, 1 mM NADH, or 1 mM NADPH. The control reactions were performed with the absence of NADH and NADPH. After 1-hour incubation at 28°C, reaction products were extracted with ethyl acetate. After being dried with a vacuum concentrator, the samples were resuspended with 0.1 ml of 80% methanol, and 1 μl of sample was injected to a UHPLC-MS system with the same setting described as above. For quantification of the products, the UV absorption peak area of a particular compound from each sample was normalized to that of the internal standard (chrysin) and calibrated using a standard curve of *p*-coumaric acid or coniferaldehyde (for 5-hydroxyconiferaldehyde).

### Statistical analysis

Statistical analysis in each required experiment was performed using either Student’s *t* test with Microsoft Excel (two-tailed distribution and two-sample unequal variance) or analysis of variance (ANOVA) test with GraphPad Prism version 4 (*P* < 0.05, one-way ANOVA and Tukey’s test). Details of statistical analyses, including sample sizes and biological replications, are provided in the figure legends.

### Accession numbers

The *A. thaliana* genes are under the following The Arabidopsis Information Resource accession numbers: *AtCB5A* (At1g26340), *AtCB5B* (AT2G32720), *AtCB5C* (At2g46650), *AtCB5D* (At5g48810), *AtCB5E* (At5g53560), *AtC4H* (At2g30490), *AtF5H1* (At4g36220), *CBR1* (AT5G17770), *ATR1* (AT4G24520), and *ATR2*(AT4G30210).

## Notes

View/request a protocol for this paper from *Bio-protocol*.
