# Supplementary material for: Tissue-preferential recruitment of electron transfer chains for cytochrome P450-catalyzed phenolic biosynthesis
Source: Sci Adv. 2023 Jan 11;9(2):eade4389. doi: 10.1126/sciadv.ade4389 (PMC9833660; doi:10.1126/sciadv.ade4389)
Supplement: Supplementary file 1 — Figs. S1 to S15 Table S1 [file sciadv.ade4389_sm.pdf]

Science Advances
AAAS

Supplementary Materials for

Tissue-preferential recruitment of electron transfer chains for cytochrome P450-catalyzed phenolic biosynthesis

Xianhai Zhao *et al.*

Corresponding author: Chang-Jun Liu, cliu@bnl.gov

Sci. Adv. **9**, eade4389 (2023)
DOI: 10.1126/sciadv.ade4389

This PDF file includes:

Figs. S1 to S15
Table S1

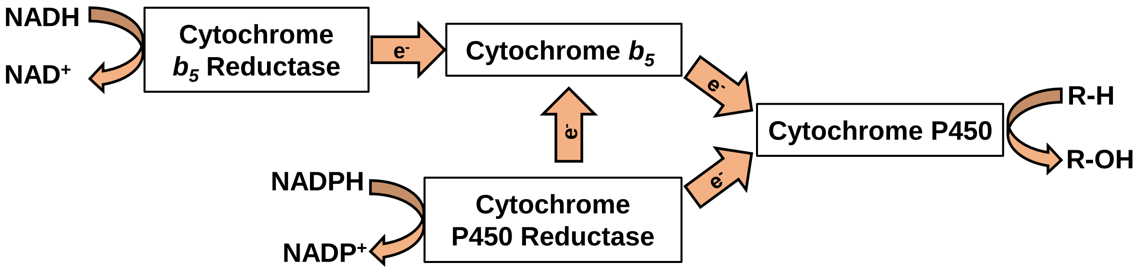

Fig. S1 Endoplasmic reticulum electron transfer chains supplying reducing equivalence for cytochrome P450s.

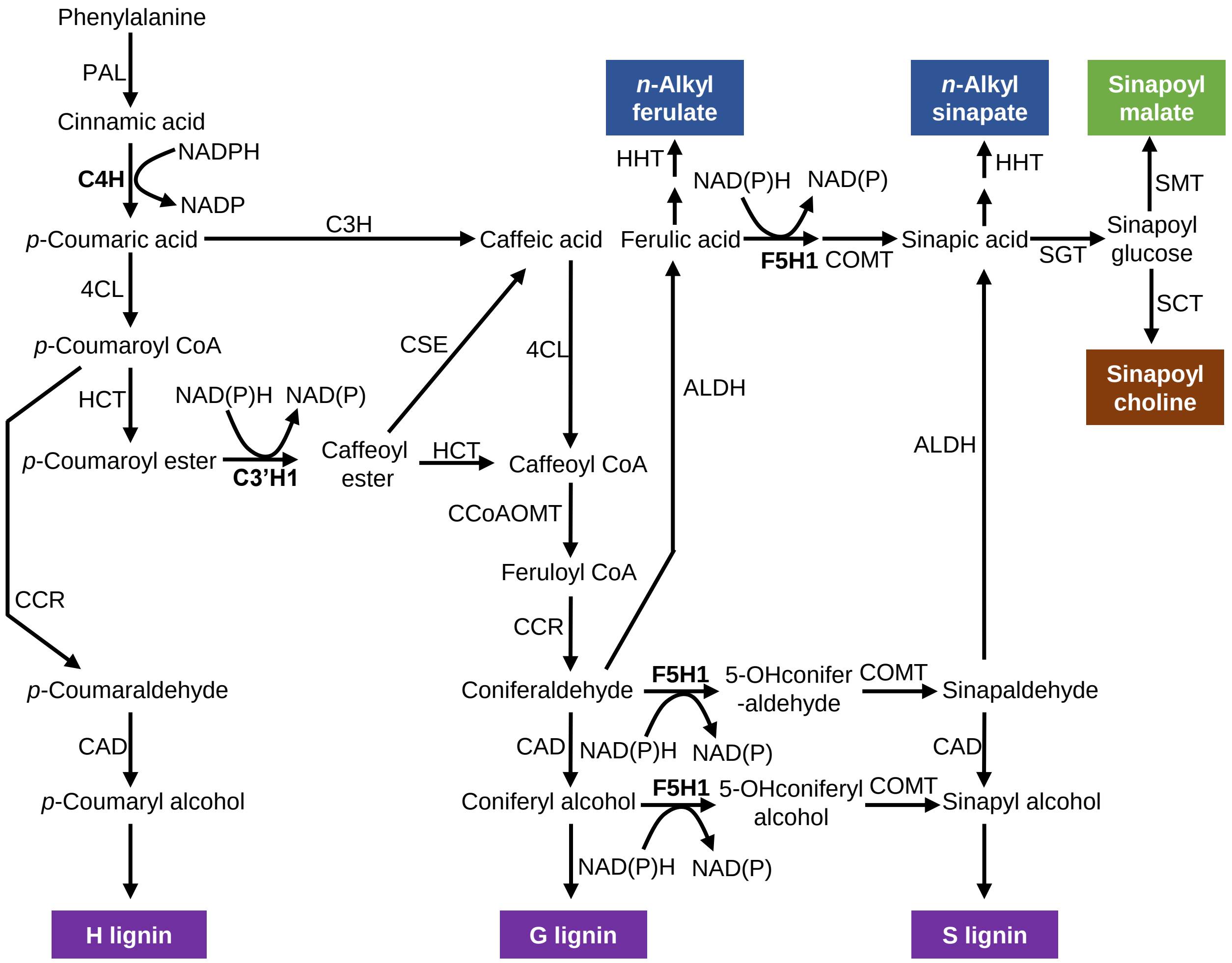

Fig. S2 The phenylpropanoid biosynthetic pathway leading to the syntheses of leaf sinapoyl malate, seed sinapoylcholine, suberin-bound phenolics and stem lignin.
ALDH, aldehyde dehydrogenase; C3H, *p*-coumarate 3-hydroxylase; C3'H1, *p*-coumaroylshikimate 3'-hydroxylase 1; C4H, cinnamic acid 4-hydroxylase; CAD, (hydroxy)cinnamyl alcohol dehydrogenase; CCoAOMT, caffeoyl CoA 3-*O*-methyltransferase; CCR, cinnamoyl CoA reductase; 4CL, 4-hydroxycinnamoyl CoA ligase; COMT, caffeic acid/5-hydroxyferulic acid *O*-methyltransferase; CSE: caffeoyl shikimate esterase; F5H1, ferulate/coniferaldehyde/coniferyl alcohol 5-hydroxylase 1; HCT, hydroxycinnamoyl CoA : shikimate/quinate hydroxycinnamoyltransferase; HHT, hydroxyfatty acid: hydroxycinnamoyl transferase; PAL, phenylalanine ammonia lyase; SCT, sinapoylglucose:choline sinapoyltransferase; SGT, sinapate glucosyltransferase; SMT, sinapoylglucose:malate sinapoyltransferase.

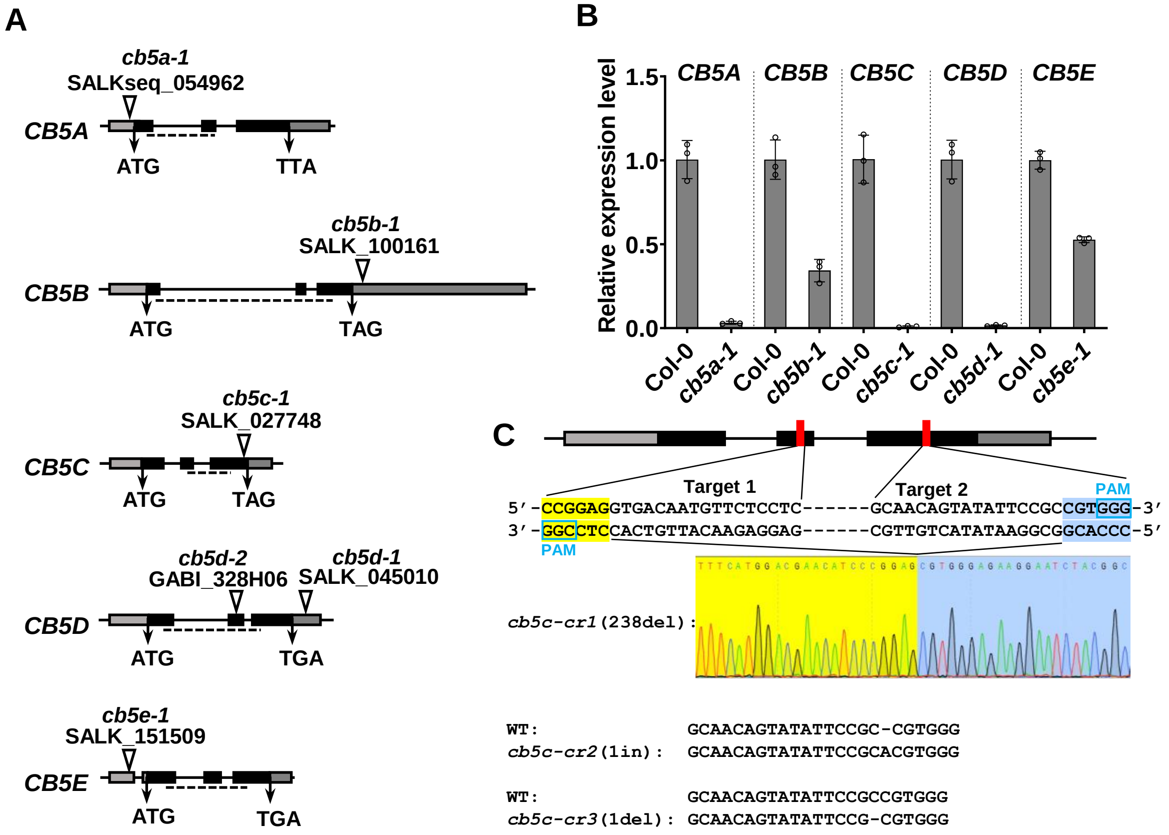

Fig. S3 Characterization of *cb5* alleles.

(**A**) Structures of the *CB5A, CB5B, CB5C, CB5D* and *CB5E* genes, showing the position of T-DNA insertions. The dashed lines show the regions used for qRT-PCR analysis. (**B**) The relative transcript abundance of *CB5* genes in 7-day-old seedlings of the wild type (Col-0) and *cb5* mutants. The expression level is normalized to that of *PP2A* gene then presented relative to that of Col-0 wild-type. Data are presented as means ± s. d. of three biological replicates. (**C**) CRISPR/Cas 9 gene editing strategy to generate *cb5c-cr* mutants. Two CRISPR targets and their sequences are shown. Sanger sequencing chromatogram and/or sequences indicate the 238 bp deletion, 1 bp insertion and 1 bp deletion in the obtained *cb5c-cr* mutants.

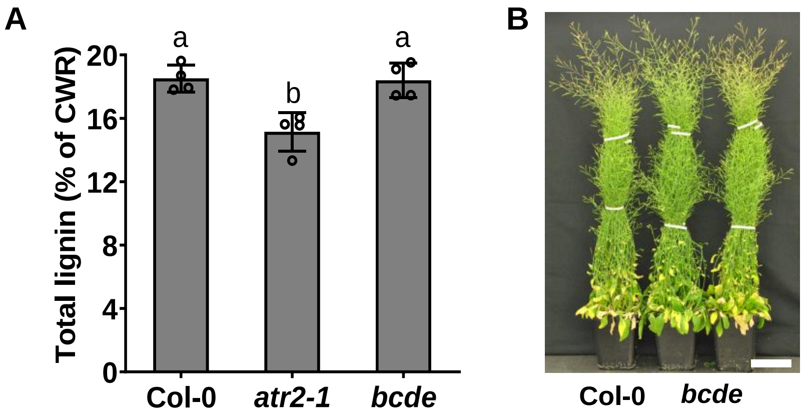

Fig. S4 Total lignin content and plant growth phenotypes of *cb5* quadruple mutants.

(**A**) Total acetyl bromide lignin content in the cell walls of 11-week-old plants of the Col-0, *atr2-1* and *bcde*. Data are presented as means ± s.d. of four biological replicates. Letters above the bars indicate significant differences ($P < 0.05$), determined by one-way ANOVA test. CWR: Cell wall residue. (**B**) Growth phenotypes of 9-week-old plants of the Col-0 wild-type and *cb5* quadruple mutants. Scale bar = 10 cm.

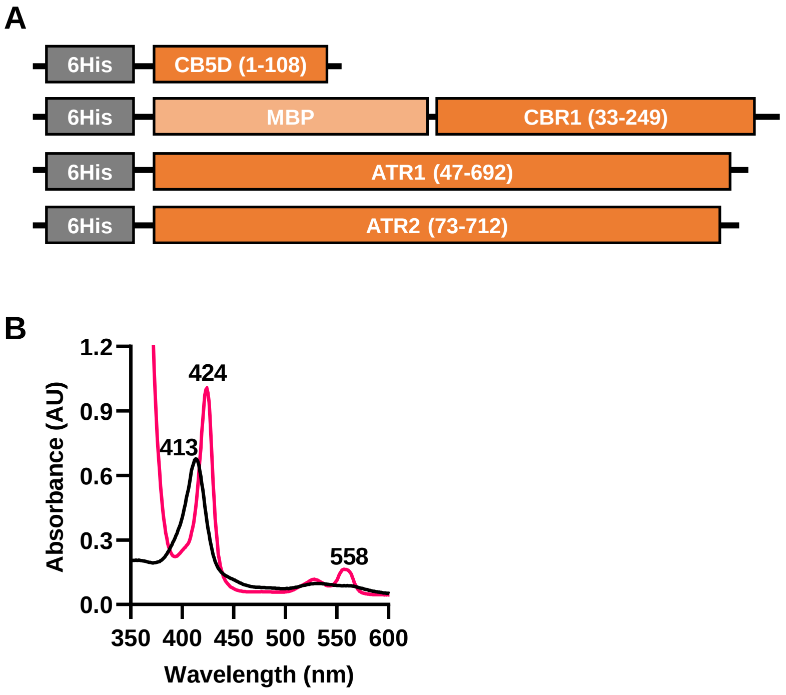

Fig. S5 The recombinant proteins of the truncated CB5D, CBR1, ATR1 and ATR2. (**A**) The schema of fusion proteins of the truncated CB5D (residues 1-108), CBR1 (residues 33-249), ATR1 (residues 47-692) and ATR2 (residues 73-712), in which the predicted transmembrane domain of each protein was deleted. (**B**) Absolute absorption spectra of the purified recombinant CB5D in the oxidized form (black line) and reduced form after reduction by dithionate (red line). AU, absorbance units.

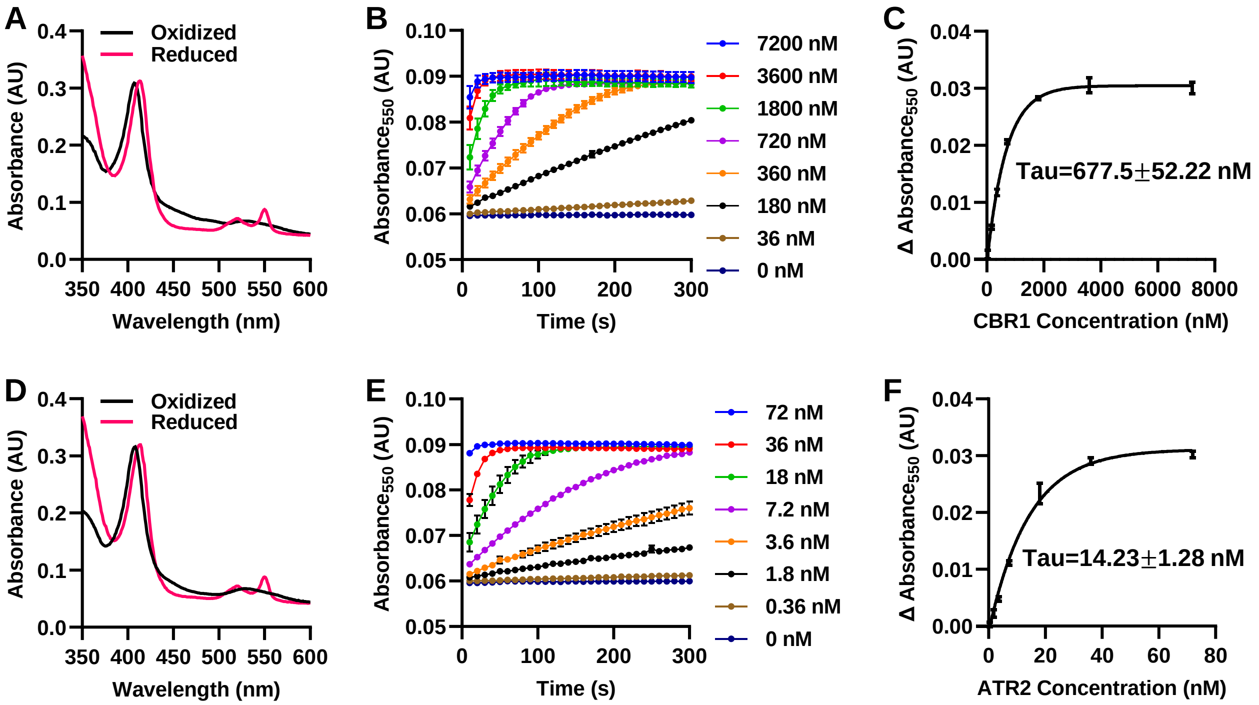

Fig. S6 Reduction of Cytochrome C by CBR1 and ATR2.
Cytochrome C (Cyt C) (7 µM) was reduced by different concentrations of CBR1 (**A** to **C**) or ATR2 (**D** to **F**) at room temperature. The reaction was initiated by addition of either 100 µM NADH (**A** to **C**) or 100 µM NADPH (**D** to **F**) as electron donor. (**A**) and (**D**) Absolute absorption spectra of the oxidized and the reduced Cyt C protein before and after incubation with 7200 nM CBR1 (**A**) or 72 nM ATR2 (**D**) for 10 mins. (**B**) and (**E**) The changes of absorbance at 550 nm of Cyt C over the indicated reaction period with different concentrations of CBR1 (**B**) or ATR2 (**E**). The absorbance was recorded for 30 cycles with 10 s interval. (**C** and **F**) The monophasic kinetics of Cyt C reduction by CBR1 (**C**) or ATR2 (**F**) at the point of 60 second reaction at the function of different concentrations of the reductase. Data are presented as means ± s.d. of three independent experiments. Tau is the enzyme concentration constant. AU, absorbance units.

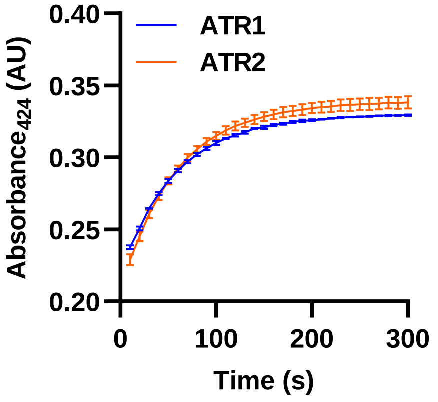

Fig. S7 Reduction of CB5D by ATR1 and ATR2.

The recombinant CB5D (7 µM) was incubated with 720 nM ATR1 or ATR2 at room temperature for the indicated time. The reactions were initiated with addition of 100 µM NADPH and the absorbance at 424 nm were recorded with a microplate reader for 30 cycles with 10 s interval. Data are presented as means ± s.d. of three independent experiments. AU, absorbance units.

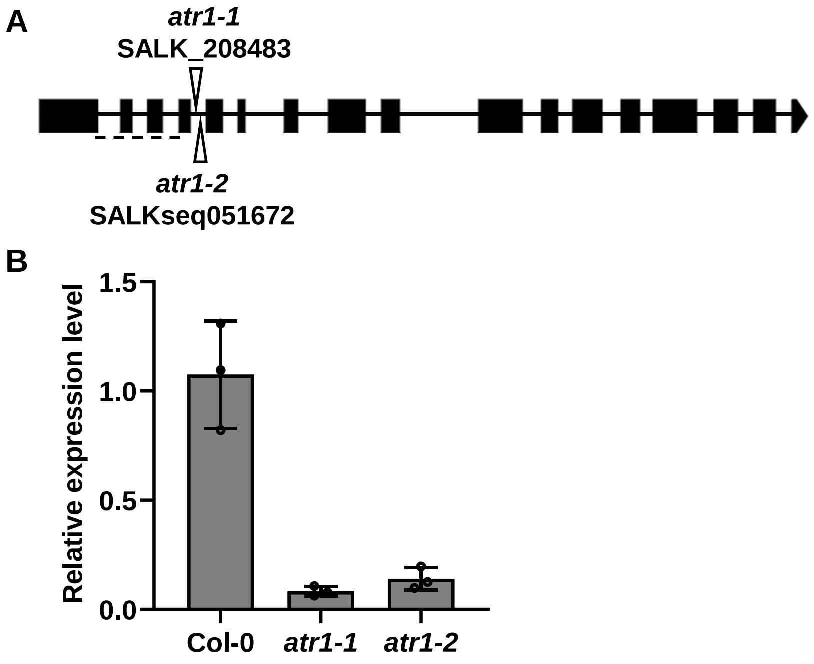

Fig. S8 Characterization of *atr1-1* and *atr1-2* mutant.
(**A**) Structure of the *ATR1* genes. Blank triangles show the positions of T-DNA insertions in two mutant alleles. The dashed line shows the region used for qRT-PCR analysis. (**B**) Transcript abundance of *ATR1* gene in 7-day-old seedlings of the Col-0 wild-type, *atr1-1* and *atr1-2* mutants. The expression levels are normalized to that of *PP2A* gene and then to that of Col-0 wild-type. Data are presented as means ± s.d. of three biological replicates.

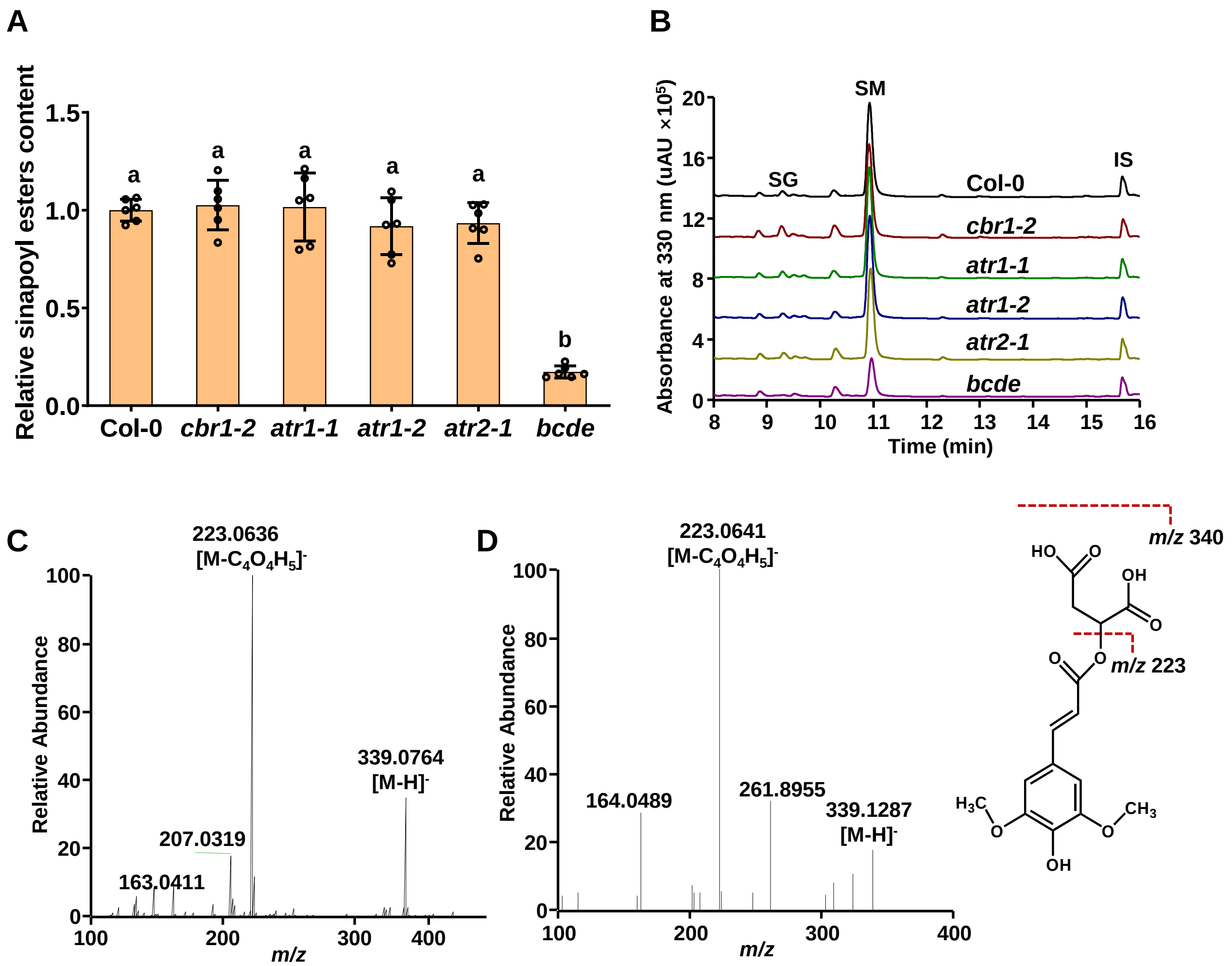

Fig. S9 Accumulation of leaf sinapoyl esters in the redox component mutants.
(**A**) Relative sinapoyl ester contents in the 4-week-old rosette leaves of Col-0 wild-type, *cbr1-2, atr1-1, atr1-2, atr2-1* and *bcde* mutants. The content in Col-0 wild-type was set as 1. Two rosette leaves from different plants were mixed representing one biology replicate. Data are presented as means ± s.d. of six biological replicates. Letters above the bars indicate significant differences ($P < 0.05$), determined by one-way ANOVA test. (**B**) UHPLC-UV profiles of sinapoyl esters in the 4-week-old rosette leaves of the indicated genotypes. SG, sinapoyl glucose; SM, sinapoyl malate; IS, chrysin as internal standard; AU, absorbance units. (**C** and **D**) MS and MS_2 spectra of sinapoyl malate.

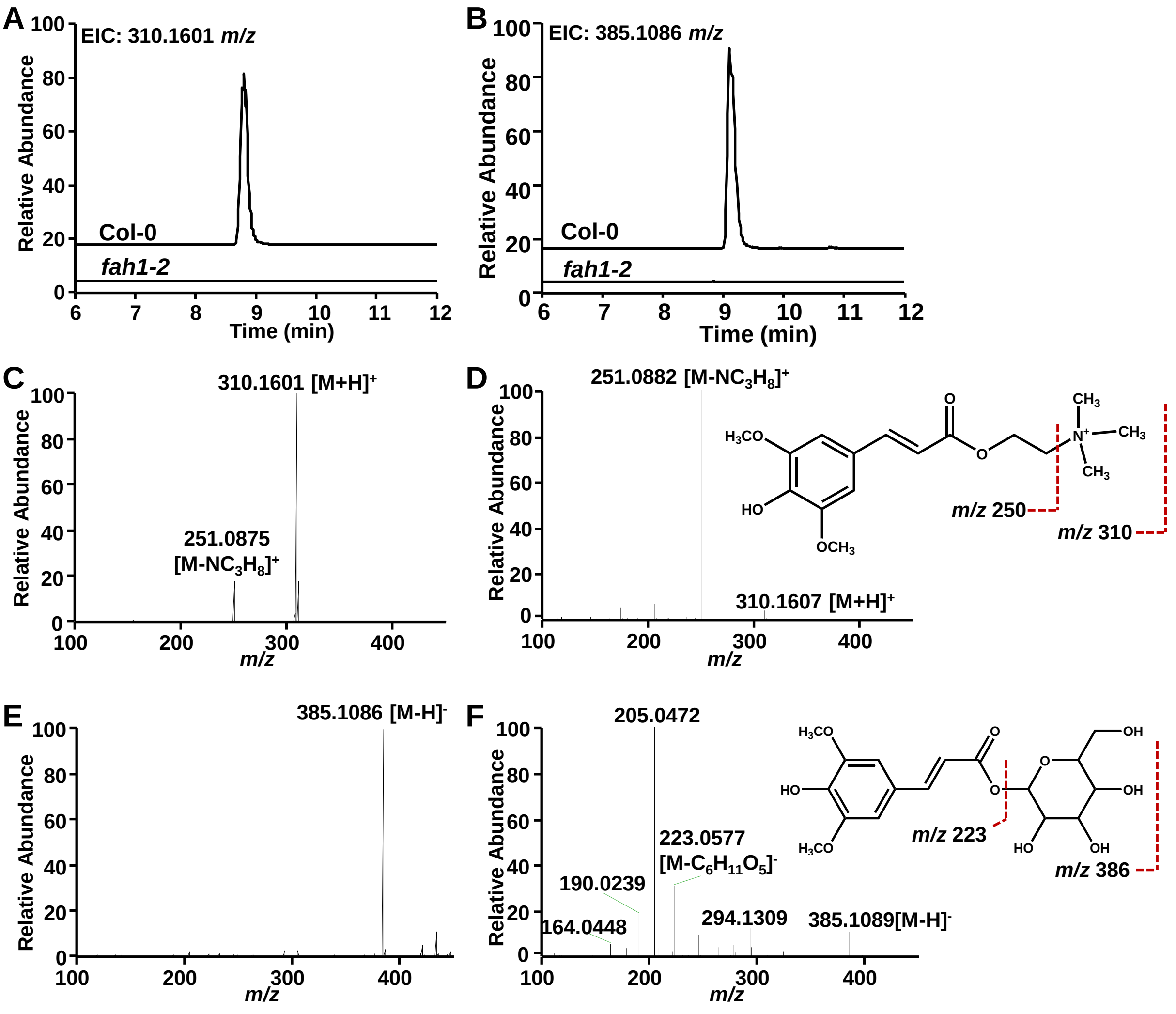

Fig. S10 UHPLC-MS analysis of seed sinapoyl esters.

(**A**) and (**B**) UHPLC-MS extracted ion chromatograms (EICs) of seed sinapoylcholine (at *m/z* of 310.1601) and sinapoyl glucose (at *m/z* of 385.1086) in Arabidopsis wild type (Col-0) and *fah1-2* mutant. (**C** to **F**) MS and MS_2 spectra of sinapoylcholine (**C** and **D**) and sinapoyl glucose (**E** and **F**).

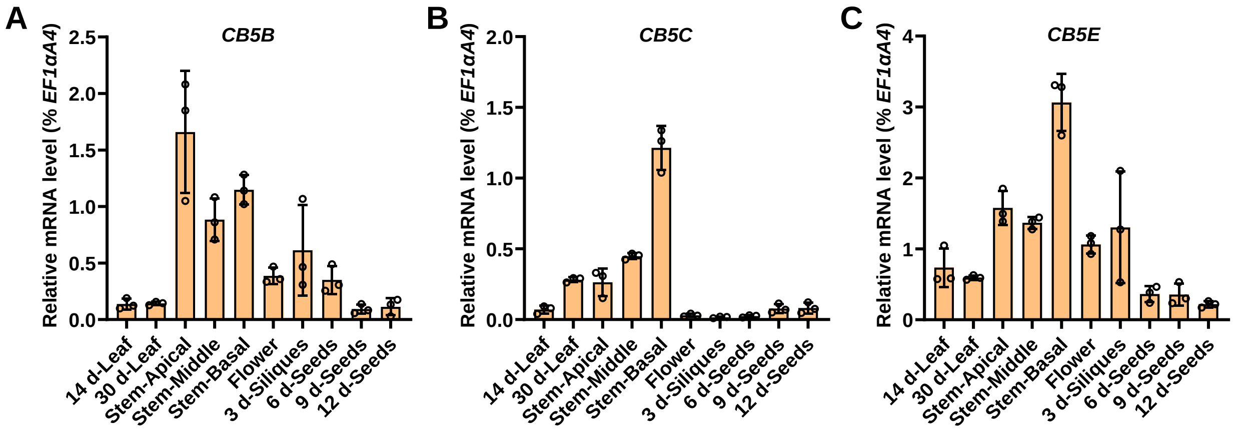

Fig. S11 RT-qPCR quantification of the transcript abundance of CB5 genes.
Relative transcript abundance of *CB5B* (**A**), *CB5C* (**B**) and *CB5E* (**C**) in the indicated *Arabidopsis* tissues was quantified by RT-qPCR. The leaves were from 14-day-old seedlings and 30-day-old plants; the stems and flowers were from 50-day-old plants; the siliques and seeds were collected at the indicated day post anthesis. Tissues from at least five individual plants were pooled as one biological replicate. The expression levels were normalized to that of *EF1αA4* gene. Data are presented as means ± s.d. of three biology replicates.

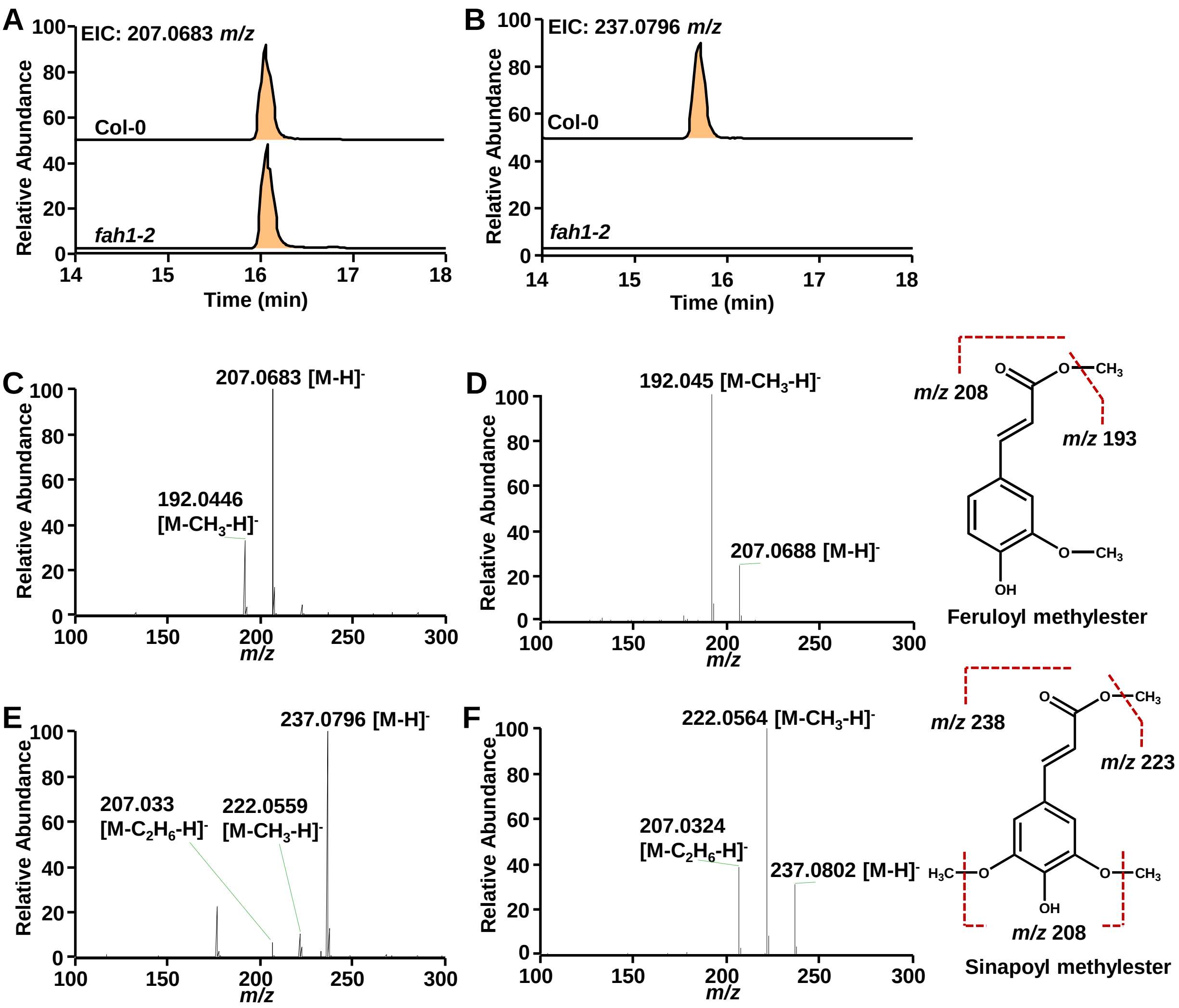

Fig. S12 LC-MS analysis of seed coat suberin aromatics.

(**A**) and (**B**) UHPLC-MS extract ion chromatography of ferulate methylester (at *m/z* 207.0683) and sinapate methylester (at *m/z* 237.0796) released from the Col-0 wild-type and *fah1-2* mutant. (**C** to **F**) MS and MS_2 spectra of ferulate methylester (**C** and **D**) and sinapate methylester (**E** and **F**).

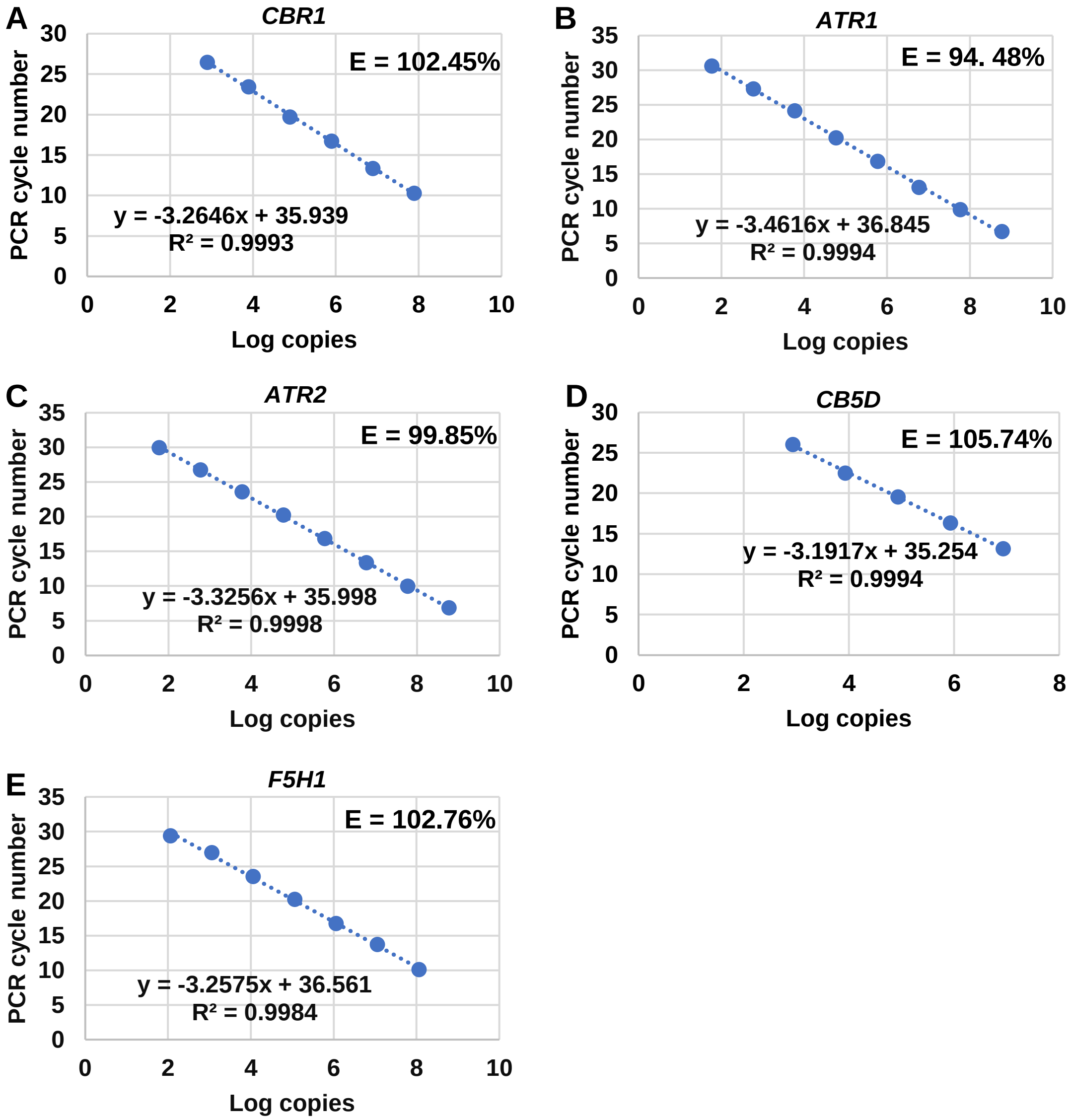

Fig. S13 The qPCR efficiency of the designed primers for *CBR1* (A), *ATR1* (B), *ATR2* (C), *CB5D* (D) and *F5H1* (E) genes.

A series of diluted DNA standards of the redox component gene in pDONR207 (1: 10 dilutions with three replicates each) were used as PCR templates. Three replication were performed for every sample. E: Amplification efficiency.

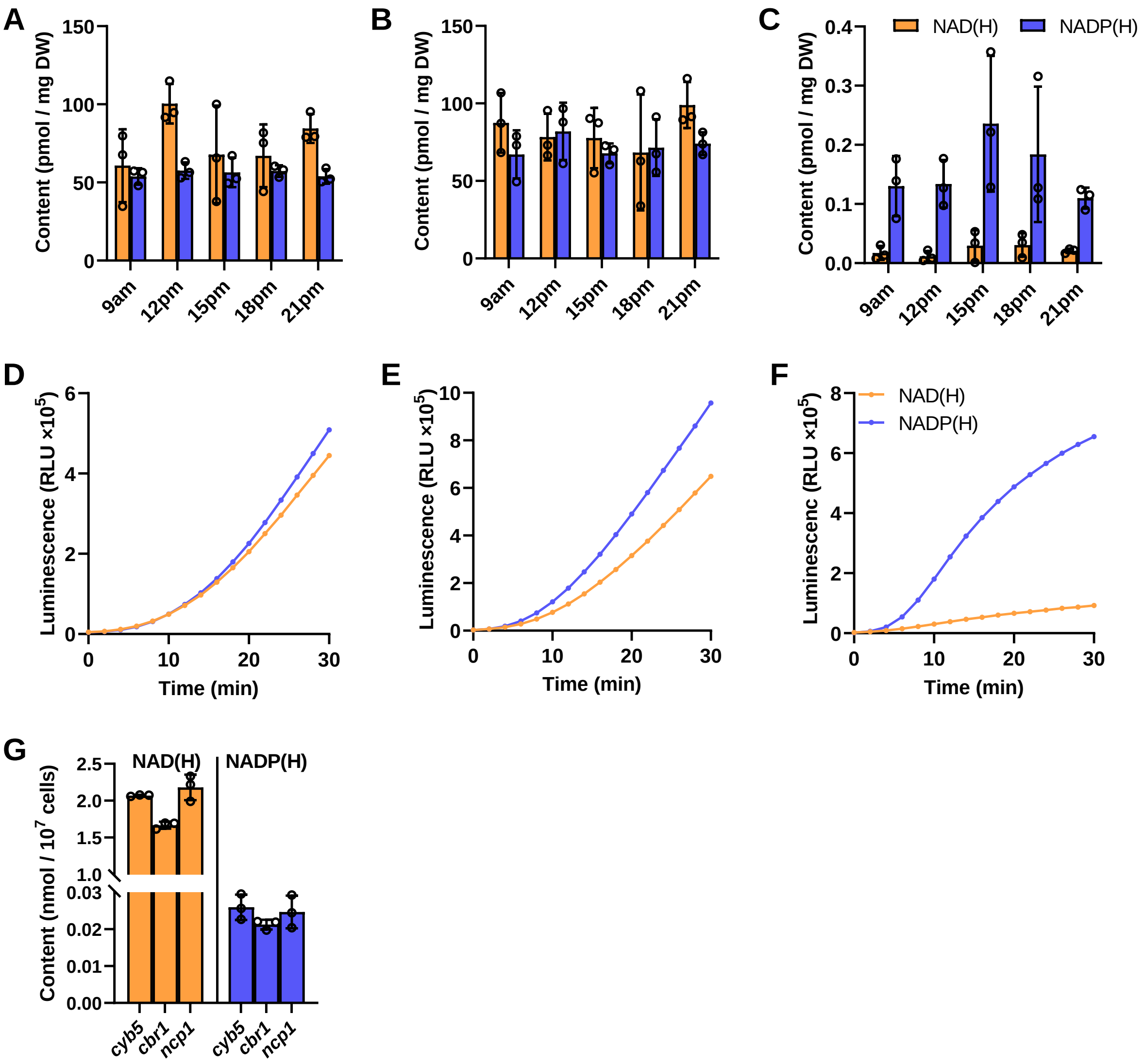

Fig. S14 Quantification of NAD(H) and NADP(H) levels in Arabidopsis and yeast.
(**A-C**) The calculated absolute amount of NAD(H) and NADP(H) and (**D-F**) the representative detection profiles of the luminescence signals of NAD(H) (orange) and NADP(H) (blue), detected with NAD(H)- and NADP(H)-Glo assay kits, in the rosette leaves from 30-day-old Arabidopsis (**A** and **D**), developing seeds from 9- to 12-day-post anthesis siliques (**B** and **E**), and developing stems from 42-day-old plants (**C** and **F**). Arabidopsis plants were grown in a growth chamber with 16 hr light (from 8 am to 24 pm) / 8 hr dark (from 0 am to 8 am) regime. The materials were sampled at the indicated time points with 3 hr intervals (**A-C**). The luminescence signals were recorded for 15 cycles with 2 min intervals over the indicated 30 min time range with a microplate reader and the luminescence intensity at 30 min was used to calculate the absolute NAD(H) or NADP(H) content with their respective standard curves. DW, dry weight. RLU, relative light units. (**G**) The absolute contents of NAD(H) and NADP(H) detected in *cyb5*, *cbr1* and *ncp1* yeast cells. Data are presented as means ± s.d. of three biology replicates in (**A-C** and **G**).

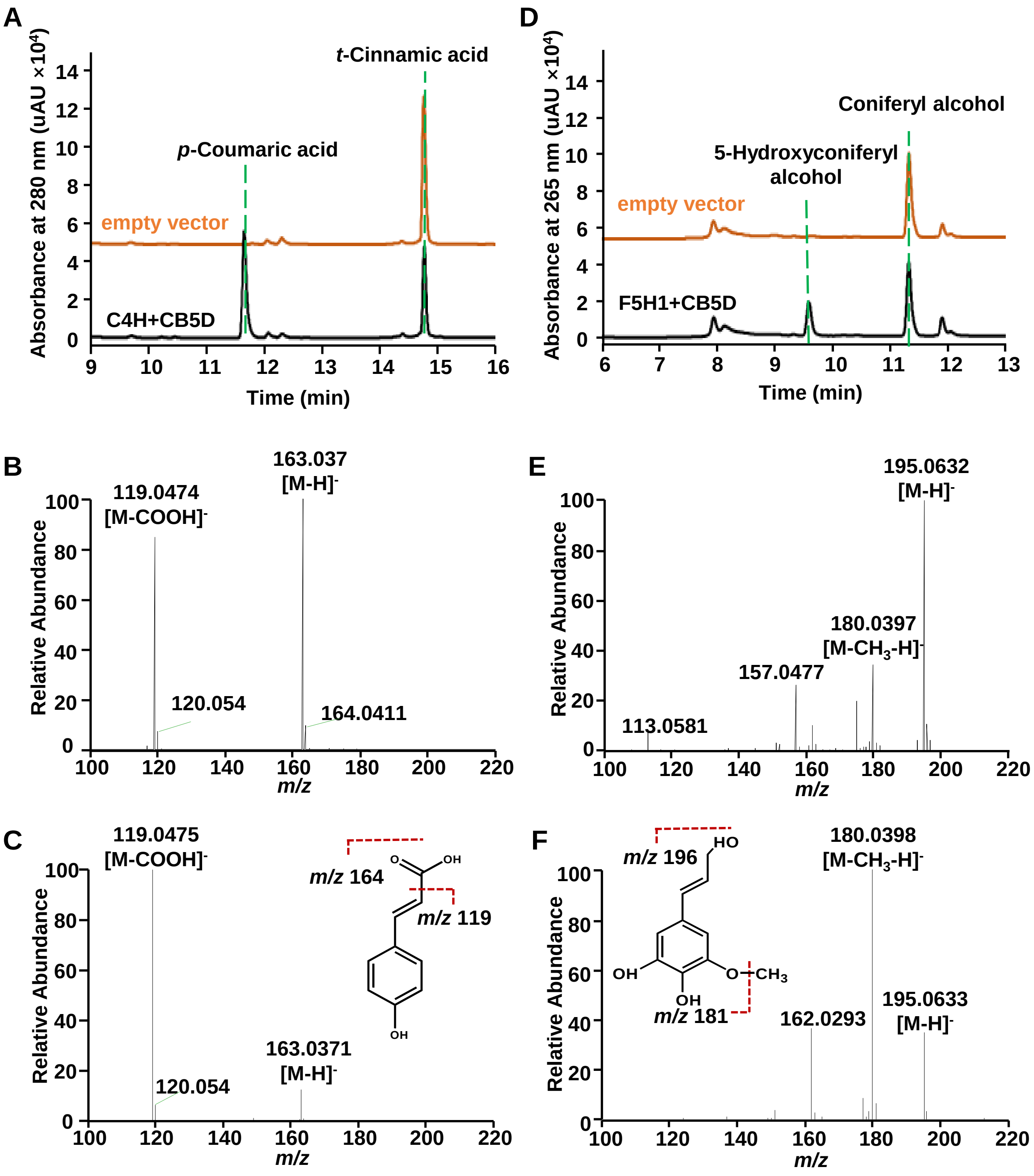

Fig. S15 Whole-cell biocatalytic assays for C4H and F5H1 catalytic activities.
(**A**) UHPLC-UV profile of C4H-catalyzed biotransformation of *t*-cinnamic acid to *p*-coumaric acid within the *cyb5* yeast strain harboring Arabidopsis CB5D gene. (**B**) Mass spectrum of the obtained *p*-coumaric acid in C4H-catalyzed biotransformation. AU, absorbance units. (**C**) MS_2 spectrum of the molecular ion at *m/z* of 163.037 in (**B**). (**D**) UHPLC-UV profile of F5H1-catalyzed biotransformation of coniferyl alcohol to 5-hydroxyconiferyl alcohol within the *cyb5* yeast strain harboring Arabidopsis CB5D gene. (**E**) Mass spectrum of the obtained 5-hydroxyconiferyl alcohol in F5H1-catalyzed reaction. (**F**) MS_2 spectrum of the molecular ion at *m/z* of 195.0632 *m/z* in (**E**). Experiments were performed three times independently.

Table S1. Primers used in this study.

Primer's name	Primer sequences (5'-3')
**qRT-PCR**	
AtCB5A-F	AGCAAGATGACTGCTGGGTC
AtCB5A-R	GCGACAGCAAGAAGCACATC
AtCB5B-F	AGCTCATGACTGTTGGATTGTC
AtCB5B-R	CTGTGACCCACGTCCTCAAA
AtCB5C-F	ACATCCCGGAGGTGACAATG
AtCB5C-R	CGCCGTAGATTCCTTCTCCC
AtCB5D-F	ACAGTAGCGCCAAGGATTGT
AtCB5D-R	ATCGGTCGCATCTTTCCCTG
AtCB5E-F	TGATCATCCTGGAGGCGATG
AtCB5E-R	TCCATCATGTCCCTTGCAGT
ATR1-F	GGATTTGCTAAGGCATTA
ATR1-R	CTCCATAAGTAGCAACAC
ATR2-F	GATGATTACGCGGCTGATG
ATR2-R	GTCGGTAGGCTCACCATCTC
CBR1-F	GAGATGCGTGTTGGAGACCA
CBR1-R	TGGAACATGGGAGTGATGCC
F5H1-F	GGTCTCTTGTAACGTTGGTAAGCC
F5H1-R	ACGCTGCCCGGTAAGTTATGTTG
PP2A-F	TATCGGATGACGATTCTTCGTGCAG
PP2A-R	GCTTGGTCGACTATCGGAATGAGAG
EF1αA4-F	CTGGAGGTTTTGAGGCTGGTAT
EF1αA4-R	CCAAGGGTGAAAGCAAGAAGA
**Genotyping**	
cb5a-1-LP	TATCTGGCCATGGAGTAGCAG
cb5a-1-RP	AGCCATATCATTTTCTTGGGG
cb5b-1-LP	CACACGACAACGTTTTGAATG
cb5b-1-RP	TCAGAAGTGGATCTTCCCATG
cb5c-1-LP	AAACATAACGCGTGTGGTCTC
cb5c-1-RP	AAGTATGCCCTCACCC
cb5d-1-LP	TTGGTGCTGCTTAAGATGTCTC
cb5d-1-RP	TGTCCCCCAAAGACAACTATG
cb5d-2-LP	TGTCCCCCAAAGACAACTATG
cb5d-2-RP	TTGGTGCTGCTTAAGATGTCTC
cb5e-1-LP	CGGTTTGATATGTGGAGATGG
cb5e-1-RP	CTACGAGGGGCTCGATTAAAG
atr1-1/2-LP	TGATTCTTTCCTGCAAACCAC
atr1-1/2-RP	TTATCCGAAGAAATCAAAGCG
LB1.3	ATTTTGCCGATTTCGGAAC
GABI_8409	ATATTGACCATCATACTCATTGC
**Plasmid constructs**	
**pHEE401E-CB5C**	
DT1-CB5C-BsF	AACGAGGTGACAATGTTCTCCTCAATCTCTTAGTCGACTCTAC
DT1-CB5C-F0	ATTATTGGTCTCGAAACGAGGTGACAATGTTCTCCTC
DT2-CB5C-R0	ATATATGGTCTCGATTGCAACAGTATATTCCGCCGTGTT
DT2-CB5C-BsR	TGCAACAGTATATTCCGCCGTGTTTTAGAGCTAGAAATAGC

**pYeDP60-F5H1-T2A-CB5D-P2A-C4H**	
F5H1-F	AATACACACACTAAATTACCGGATCCATGGAGTCTTCTATATCACAAAC
F5H1-R	CTCCTCCACGTCACCGCATGTTAGAAGACTTCCTCTGCCCTCAAGAGCACAGATGAGGCG
CB5D-F	GTCTTCTAACATGCGGTGACGTGGAGGAGAATCCCGGCCCTATGGGCGGAGACGGAAAAG
CB5D-R	CTCCAGCTTGCTTAAGAAGAGAAAAATTAGTAGCTCCAGATCCAGAAGAAGGAGCCTTGG
C4H-F	TCTTCTTAAGCAAGCTGGAGATGTTGAAGAAAATCCTGGACCCATGGACCTCCTCTTGCT
C4H-R	AGACATGGGAGATCCCCCGCGAATTCTTAACAGTTCCTTGGTTTCATAAC
**pYEDP60-F5H1-T2A-CB5D-P2A-C4H-T2A-CBR1/ATR1/ATR2**	
F5H1-F	AATACACACACTAAATTACCGGATCCATGGAGTCTTCTATATCACAAAC
F5H1-R	CTCCTCCACGTCACCGCATGTTAGAAGACTTCCTCTGCCCTCAAGAGCACAGATGAGGCG
CB5D-F	GTCTTCTAACATGCGGTGACGTGGAGGAGAATCCCGGCCCTATGGGCGGAGACGGAAAAG
CB5D-R	CTCCAGCTTGCTTAAGAAGAGAAAAATTAGTAGCTCCAGATCCAGAAGAAGGAGCCTTGG
C4H-F	TCTTCTTAAGCAAGCTGGAGATGTTGAAGAAAATCCTGGACCCATGGACCTCCTCTTGCT
C4H-R	CTCCACGTCACCGCATGTTAGAAGACTTCCTCTGCCCTCACAGTTCCTTGGTTTCATAA
CBR1-F	CTAACATGCGGTGACGTGGAGGAGAATCCCGGCCCTATGGATACCGAGTTTCTC
CBR1-R	AGACATGGGAGATCCCCCGCGAATTCTCAGAACTGGAATTGCATC
ATR1-F	CTAACATGCGGTGACGTGGAGGAGAATCCCGGCCCTATGACTTCTGCTTTGTATGCTTC
ATR1-R	AGACATGGGAGATCCCCCGCGAATTCTCACCAGACATCTCTGAGGTATC
ATR2-F	CTAACATGCGGTGACGTGGAGGAGAATCCCGGCCCTATGTCCTCTTCTTCTTCTTC
ATR2-R	AGACATGGGAGATCCCCCGCGAATTCTTACCATACATCTCTAAGATATC
**pDONR207-CB5s**	
attB1-CB5A	GGGGACAAGTTTGTACAAAAAAGCAGGCTGCATGCCGACACTCACAAAGCT
attB2-CB5A	GGGGACCACTTTGTACAAGAAAGCTGGGTCTTAAGTCTTGCGAGAGAACAAGACAC
attB1-CB5B	GGGGACAAGTTTGTACAAAAAAGCAGGCTGCATGGGAGACGAAGCAAAG
attB2-CB5B	GGGGACCACTTTGTACAAGAAAGCTGGGTCCTACCCTGATTTGGTGTAGA
attB1-CB5C	GGGGACAAGTTTGTACAAAAAAGCAGGCTGCATGGCGAATCTAATTTCGTTTC
attB2-CB5C	GGGGACCACTTTGTACAAGAAAGCTGGGTCCTACTTGTTGTTGTAGAATCTG
attB1-CB5D	GGGGACAAGTTTGTACAAAAAAGCAGGCTGCATGGGCGGAGACGGAAAAG
attB2-CB5D	GGGGACCACTTTGTACAAGAAAGCTGGGTCTCAAGAAGAAGGAGCCTTGGT
attB1-CB5E	GGGGACAAGTTTGTACAAAAAAGCAGGCTGCATGTCTTCAGATCGGAAGGT
attB2-CB5E	GGGGACCACTTTGTACAAGAAAGCTGGGTCCTAGTCTTTCTTGGTATAGTG
**Prokaryotic expression**	
pET28a-CB5D-F	AGCAAATGGGTCGCGGATCCGAATTCATGGGCGGAGACGGAAAAG
pET28a-CB5D-R	CAGTGGTGGTGGTGGTGGTGCTCGAGTCAATCCGAGCTCTTATCCTGAG
pET28a-ATR1-F	AGCAAATGGGTCGCGGATCCGAATTCATGTGGAAGAAAACGACGGCGG
pET28a-ATR1-R	CAGTGGTGGTGGTGGTGGTGCTCGAGTCACCAGACATCTCTGAGGTATC
pET28a-ATR2-F	AGCAAATGGGTCGCGGATCCGAATTCATGAGGAGATCCGGTTCTGGGAA
pET28a-ATR2-R	CAGTGGTGGTGGTGGTGCTCGAGTTACCATACATCTCTAAGATATCTTCC
pMAL-C2X-CBR1-F	GGATCCCCGGAATTCACATCCTCCAAGAAACGCAG
pMAL-C2X-CBR1-R	CTGCAGGTCGACTCAGAACTGGAATTGCATCTCC
pET28a-MBP-F	GCAAATGGGTCGCGGATCCGAATTGCATATGAAAATCGAAGAAGG
pET28a-CBR1-R	GTGGTGGTGGTGGTGCTCGAGTCAGAACTGGAATTGCATCTC
